# An MRI-derived head-neck finite element model

**DOI:** 10.1007/s10237-025-02013-x

**Published:** 2025-10-03

**Authors:** Hossein Bahreinizad, Gustavo M. Paulon, Leonardo Wei, Suman K. Chowdhury

**Affiliations:** 1 Department of Industrial and Systems Engineering, University of Florida, Gainesville, FL USA; 2 Department of Industrial, Manufacturing and Systems Engineering, Texas Tech University, Lubbock, TX USA

**Keywords:** Simulation and modeling, Finite element method, Traumatic brain injury, Computational biomechanics, Neck contribution, Image processing

## Abstract

This study aimed to develop and validate a magnetic resonance imaging (MRI)-derived biofidelic head-neck finite element (FE) model comprised of scalp, skull, CSF, brain, dura mater, pia mater, cervical vertebrae, and disks, 14 ligaments, and 42 neck muscles. We developed this model using head and neck MRI images of a healthy male participant and by implementing a novel brain hexahedral meshing algorithm and a scalp erosion model. The model was validated by replicating three experimental studies: Alshareef’s brain sonomicrometry study, NBDL's high-acceleration profile, and Ito’s frontal impact cervical vertebrae study. The results also showed that the segmented geometries of the model aligned closely with the literature data (within 3 $\sigma$ limit). The brain displacement results of the model aligned well (*r* = 0.48–0.96) with those reported in Alshareef’s experimental study. The head-neck kinematic responses of the model showed a strong correlation (*r* > 0.97) with the NBDL’s experimental results. The simulation of Ito’s experimental condition yielded peak shear strain values of the cervical spine within 1 $\sigma$ of the experimental data. Our developed head-neck FE model provides an effective computational platform for advancing brain and head injury biomechanics research and evaluating protective equipment in various impact scenarios.

**Supplementary Information:**

The online version contains supplementary material available at 10.1007/s10237-025-02013-x.

## Introduction

In the field of biomechanics, computational head-neck models based on finite element (FE) methods play a pivotal role in assessing the responses of the brain and other head-neck components to various impact scenarios (Bayly et al. 2021; Giudice et al. 2019). These scenarios encompass a wide range, from contact sports (Sahler and Greenwald 2012) and motor vehicle accidents (Teo et al. 2007) to battlefield scenarios (Zhang et al. 2013) and occupational settings (Wu et al. 2017). FE modeling is an invaluable tool in understanding the spatial and temporal distribution of the impact stress and whether they exceed the strength and deformation tolerance limits of the constituent structures. This capability is especially crucial, given the challenge of measuring such responses through in-vivo experiments. While recent advancements in computational power and the availability of tissue material properties have made modeling the complex geometries of head-neck structures more accessible than ever, there remain significant challenges in the development of sophisticated and biologically accurate head-neck FE models.

One major challenge in the computational modeling of the head-neck structures arises from variability in *mechanical properties* among different tissues. For instance, the scalp exhibits high-damping characteristics and linear elastic behavior under mechanical loads (Trotta and Annaidh 2019). Skull shows a linear viscoelastic behavior with a higher stiffness in the elastic region (Fung 2013). The brain was found to display a distinctive non-linear viscoelastic behavior (Bilston 2011). Moreover, research has demonstrated that white matter exhibits a notably firmer mechanical response in contrast to gray matter (Budday et al. 2015), underscoring the critical need for separate modeling of these regions. In addition, modeling the cerebrospinal fluid (CSF) using fluid–structure interaction techniques to capture its fluid behavior significantly increases model complexity and computational cost, and thus remains a technical challenge. Consequently, the majority of the studies have modeled CSF as a nearly incompressible hyperelastic solid and employed tied or sliding contact definitions for CSF-brain and CSF-skull interfaces (Ghajari et al. 2017; Kleiven and Hardy 2002).

Besides material properties, the accuracy in *geometry generation* and the choice of *FE meshing* method (mesh type, element size, and mesh quality) are crucial to ensure accurate and consistent numerical solutions (Giudice et al. 2019). This becomes even more critical when developing a detailed head-neck model, as coupling many inaccurate mesh surfaces in a complex model can lead to singularities and divergence (Giudice et al. 2019). Additionally, an inappropriate meshing of biological structures, even for geometrically accurate ones, was found to yield poor numerical results (Giudice et al. 2019). For example, some previous studies modeled skull, brain, and CSF (Iwamoto et al. 2002; Kleiven 2007) with coarse meshing to run their models with available computing power. Such coarse meshing fails to capture important anatomical details of head structures (such as brain sulci and gyri structure or finer details of the cranium surface). Though optimal mesh sizes for these structures should be determined through mesh convergence studies, conducting such studies is time-consuming and can significantly increase computational cost. To our knowledge, only (Zhao and Ji 2019) conducted mesh convergence studies for their brain model. They re-meshed the Worcester Head Injury Model at five mesh densities (ranging from ~ 7,200 to 1,000,000 elements for the brain) and recommended using at least 202,800 elements for brain models. Nevertheless, some recent studies reported high-quality finer meshes (~ 2 mm) to represent the aforementioned complex head and brain structures (Ghajari et al. 2017; Khanuja and Unni 2020; Liang et al. 2022).

Another crucial aspect of meshing is the choice of the element type (Giudice et al. 2019). In general, the tetrahedral elements are widely used to model complex shapes like the skull, scalp, vertebrae, and intervertebral disks that are more compressible (Giudice et al. 2021; Herron et al. 2020; Liang et al. 2022). On the other hand, hexahedral elements can handle nearly incompressible materials more accurately than the tetrahedral elements (Giudice et al. 2021). Consequently, the hexahedral elements have been chosen by many prior models to obtain more accurate stress and pressure results in brain/CSF soft tissue simulations (Ghajari et al. 2017; Li et al. 2021). Nonetheless, most of these studies used a coarser hexahedral brain mesh size (2 ~ 7 mm), as finer hexahedral meshing for the brain and CSF remains challenging, especially with CT or MRI scan data of larger voxel sizes. For a better representation of the complex brain and CSF structures, a novel technique that can transform 3D structures that are derived from MRI or CT images of any voxel size to finer hexahedral mesh structures can help researchers and practitioners to generate finer brain or CSF meshes irrespective of their original voxel sizes.

Despite these challenges, many computational FE models have been developed over the years to investigate the causation and effectuation of head and brain injuries in various impact scenarios. These models differ greatly in anatomical details, ranging from low to high fidelity (Liang et al. 2022; Ruan et al. 1994) and from geometrically and mechanically simplistic (Shugar and Katona 1975) to complex (Liang et al. 2022) descriptions of various head and neck structures. Early head FE models were developed in 1970s (Shugar and Katona 1975; Ward and Thompson 1975) wherein researchers used linear elastic material properties and simplified geometries to represent complex head tissues so that they could be solved with available, limited computational power. As more material properties and computing power gradually became available, many researchers attempted to add complexities to their head models. For instance, head FE models like the Kungliga Tekniska Hogskolan Royal Institute of Technology (KTH) model (Kleiven 2007), Strasbourg University FE Head Model (SUFEHM) (Kang et al. 1997), University College Dublin brain trauma model (UCDBTM) (Horgan and Gilchrist 2003), and Worcester Head Injury Model (WHIM) (McAllister et al. 2012) have evolved into more sophisticated models with finer meshing and geometries over the course of time. The latest enhancements of these head FE models (Montanino et al. 2021; Wu et al. 2021; Zhao and Ji 2022) involve incorporating detailed brain tissue structures and complex properties, such as brain anisotropy. However, these models did not include a detailed representation of the *neck structure*.

Since the neck serves as the primary structural connection between the head and the rest of the body and provides stability, mobility, and load-bearing support to the head, omitting the detailed representation of the neck limits the accuracy of impact simulations (Hadagali et al. 2023; Jin et al. 2017; Wood et al. 2019). Consequently, several studies attempted to include various neck structures, such as cervical vertebra (Tse et al. 2015), cervical disks (Tuchtan et al. 2020), and neck muscles and ligaments (Liang et al. 2022) in their head models. However, modeling the intricate geometric and mechanical aspects of head-neck structures presents a formidable challenge, demanding significant time, labor, and advanced methodology. Only a limited number of studies have developed comprehensive head-neck finite element (FE) models, including pioneering efforts by the Global Human Body Model Consortium (Gayzik et al. 2009) and the Total Human Model for Safety (Iwamoto et al. 2002). These models typically implement a coarse meshing of the brain (average mesh size ~ 3 mm) and remain as two of the most commonly used head-neck models in the domains of motor vehicle safety and impact biomechanics research (Fahlstedt et al. 2021; Hadagali et al. 2023; Wu et al. 2019; Yang et al. 2022). Additionally, several recent research studies have reported incorporating active neck muscles into their head-neck finite element (FE) models (Barker and Cronin 2021; Liang et al. 2022). However, it should be noted that (Liang et al. 2022) and (Barker and Cronin 2021), respectively, did not include CSF and brain in their head-neck models.

Furthermore, the advancement in medical imaging techniques, such as computed tomography (CT) to image hard tissues (e.g., vertebrae, skull, etc.) and magnetic resonance imaging (MRI) to capture soft tissue (e.g., muscles, ligaments, etc.) images, has contributed to the development of biofidelic head-neck models. Some previous studies have used both CT and MRI techniques to image the irregular shapes of head and neck structures (Liang et al. 2022). Nonetheless, it could be cumbersome to avoid *alignment issues* between CT and MRI images during the 3D model development stage. In addition, CT imaging exposes human subjects to harmful *radiation* (Thurston 2010), whereas MRI uses strong magnetic fields to capture the tissues of interest without any ionizing radiation. Though MRI scanning is traditionally implemented to image soft tissues, the recent development of MRI techniques (Mastrogiacomo et al. 2019) has opened the door for high-quality imaging of hard tissues. Accordingly, some previous studies (Chen and Ostoja-Starzewski 2010; Ghajari et al. 2017; Khanuja and Unni 2020; Li et al. 2021; Zhao and Ji 2020) have developed their FE models using head MRI data. However, to our knowledge, none of their models considered the addition of neck structures.

Though head FE models based on MRI data have been developed in the past, a comprehensive head-neck FE model has remained hitherto undeveloped in the status quo, to our knowledge. Therefore, this study embarked on the effort to develop an MRI-based detailed head-neck FE model and validated its anatomical accuracy and biomechanical performance using widely-used experimental datasets. In comparison with existing head-neck FE models in the literature, the proposed head-neck model was developed with several methodological innovations. First, unlike many previous models that combine CT for osseous and MRI for soft tissue imaging, the detailed head (scalp, skull, dura and pia mater, CSF, gray and white matter) and neck (cervical spine, disks, ligaments, and muscles) structures of our model were developed using only MRI scan data from a male participant (52nd percentile by stature, 92nd percentile by weight). Second, a novel meshing approach was developed to generate an unstructured, finer hexahedral structure of the brain’s gray and white matter and the CSF (average mesh size ~ 1.68 mm). Third, an erosion model was implemented to the scalp to simulate its realistic energy absorption capability under any given mechanical impact.

## Materials and methods

### MRI-derived head-neck FE model development

**Fig. 1:**
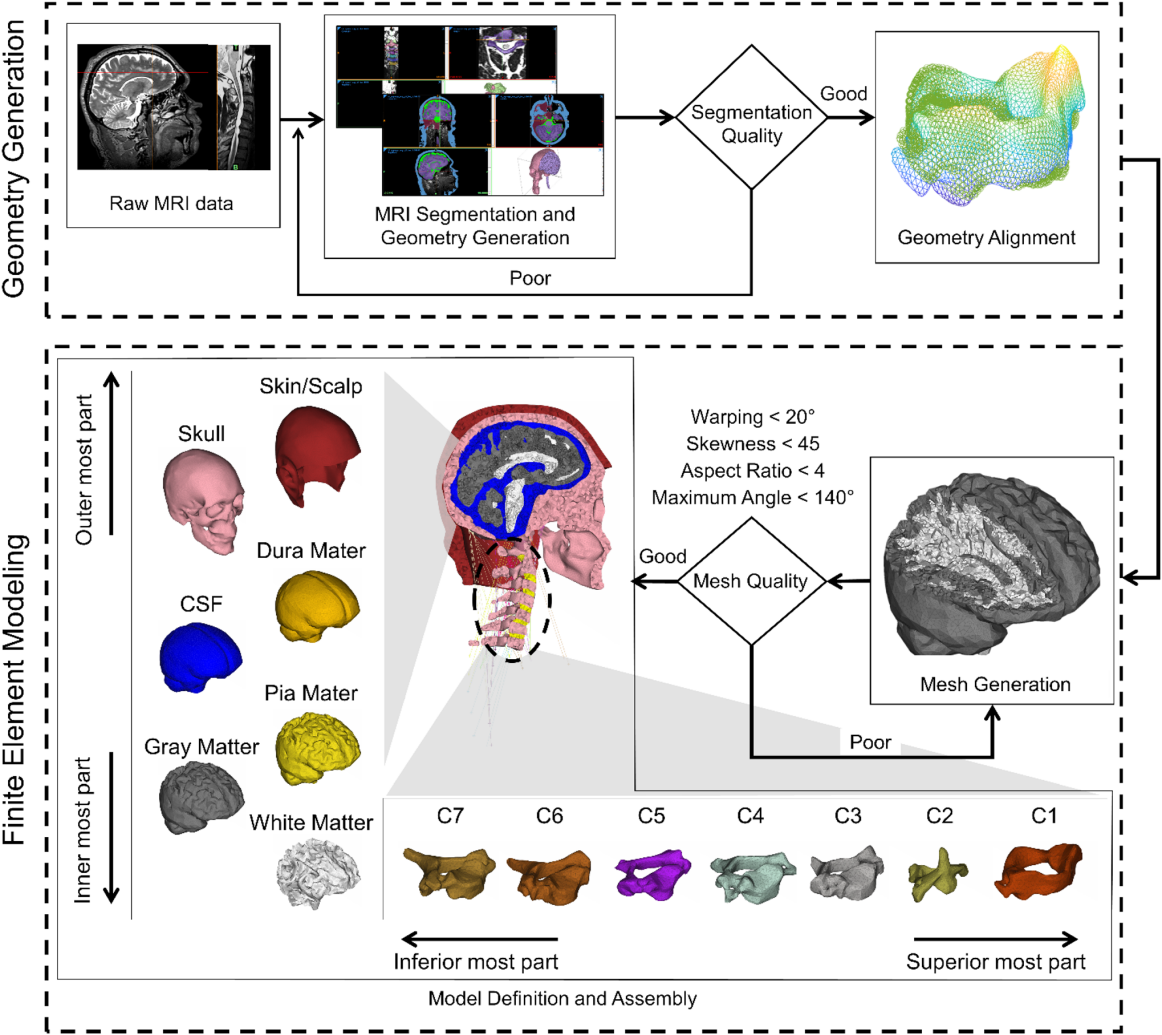
A schematic presentation of the methodological framework. The framework follows four consecutive steps: (1) 3D head-neck geometry development from a magnetic resonance imaging (MRI) dataset, (2) Geometrical verification of developed images, (3) Finite Element (FE) meshing of the model geometries, and (4) defining material properties. The steps for generating and verifying head-neck geometry are outlined in the top dashed box, and the steps for finite element modeling are provided in the bottom dashed box. Anatomical segmentation quality was assessed by comparing the geometrical dimensions of segmented components with literature-reported data, in addition to a visual inspection with respect to the subject’s raw MRI data

The methodological framework of our head-neck FE model development procedures is presented in Fig. 1. We used head and neck MRI datasets of a male firefighter (age: 42 years, height: 176 cm, weight: 106 kg, BMI: 34.2 kg/m^2^). The head anthropometric measures of the participant are presented in Table 1. Prior to the MRI procedure, we collected written informed consent from the subject. The study protocol was approved by the Texas Tech University Institutional Review Board (IRB 2020-708).

**Table 1:** The study participants’ anthropometric measures and related percentile distribution with regard to the Anthropometric Survey of U.S. Army Personnel (ANSUR II) database (Paquette 2009)

	Value	ANSUR II percentile
Stature (cm)	176.0	52nd
Weight (kg)	106.0	92nd
Head length (cm)	20.0	54th
Head circumference (cm)	57.8	62nd
Head breadth (cm)	16.0	88th

#### Head-neck geometry development

We obtained images of the head and neck structures, ranging from the top of the scalp to the third thoracic vertebra, by implementing *T*1-weighted and *T*2-weighted sequences on a 3 T MRI scanner (Siemens Medical Solutions, Germany). *T*2-weighted images were primarily used since this sequence employed different gray values to differentiate soft and hard tissues and fluids more accurately. Following image acquisition, the MRI data (except the brain and CSF) were segmented using the MIMICS 24.0 and 3-Matic 16.0 software platforms (both from Materialize Inc., Belgium). To improve segmentation accuracy, the original images that were acquired at a resolution of 2.0 mm were resliced to 0.5 mm intervals in MIMICS by using the trilinear interpolation method. Subsequently, we used identifiable masks of MIMICS to segment each head-neck structure of interest and then converted them into 3D objects. This automatic segmentation process of MIMICS may provide some inaccuracies, especially in regions near the contact surface between adjacent structures. To ensure the accuracy and fidelity of our 3D geometries, we transferred the 3D point cloud data of each structure into the 3-Matic platform to remove unwanted noise and tissues that were not eliminated during segmentation in MIMICS (Fig. 1). Where necessary, we compared our segmented geometries to their corresponding raw MRI images so that they match their original size. For the segmentation of the brain and CSF, we used MATLAB’s statistical parametric mapping (SPM) 12 toolbox (Friston et al. 1994) to segment CSF and the brain’s gray and white matter based on the International Consortium for Brain Mapping 152 atlas (Mazziotta et al. 1995).

We segmented and modeled each cervical vertebra (C1–C7) and intervertebral disk (C2–C7) based on high-resolution MRI data. The segmentation accuracy was validated against anatomical measurements reported in the literature (Table 4). The C1–C2 (atlantoaxial) joint does not contain an intervertebral disk but was modeled with detailed geometry, including proper articulation and associated ligaments and muscles. These structures were implemented to allow realistic three-dimensional (3D) rotational and translational motion.

#### Head-neck geometry verification

We used ANSA (BETA CAE Systems SA, Greece) software to directly measure geometric parameters of all 3D model geometries. The geometrical parameters were determined by following the steps, as described in previous studies (Babiloni et al. 1997; Filipek et al. 1994; Grant et al. 1987; Hagemann et al. 2008; Vasavada et al. 2008). The scalp thickness (minimum) was automatically calculated in the ANSA (BETA CAE Systems SA, Greece) software, whereas the maximum scalp thickness was chosen as the highest thickness value among the values manually measured at frontal, temporal, occipital, and parietal lobes (Hagemann et al. 2008). The average skull thickness was measured in six cranium sites (F3, F4, T3, T4, P3, P4) as specified by (Hagemann et al. 2008). Brain and CSF volumes were also measured automatically in the ANSA. The geometries of neck vertebral bodies and disks were measured by identifying four corner-most points (anterior-superior, anterior-inferior, posterior-superior, posterior-inferior points) of the vertebral body and two corner-most points (distal and proximal ends) of their spinous process in the mid-sagittal plane as described in the literature (Vasavada et al. 2008). The vertebral height was the average of anterior (distance from anterior-superior to anterior-inferior points) and posterior (distance from posterior-superior to posterior-inferior points) body heights, the vertebral depth was the average of superior (distance from posterior-superior to anterior-superior points) and inferior (distance from anterior-inferior to posterior-inferior points) vertebral body widths, spinous process length was the distance between distal and proximal corner-most points of the spinous process, and the vertebral body to spinous process length was the distance between the distal end of the spinous process and posterior side of the vertebrae body. Furthermore, we calculated angle-corrected disk heights as demonstrated in the literature (Frobin et al. 2002). We verified the accuracy of the parameters (e.g., shape, size, and volume) of individual geometries and alignments with the literature data (Babiloni et al. 1997; Filipek et al. 1994; Grant et al. 1987; Hagemann et al. 2008; Vasavada et al. 2008).

During the MRI procedure, the subject was required to lie flat and use pillows to immobilize his head and neck, which might have led to some degree of neck tilt. Consequently, we analyzed the orientation of each cervical vertebra relative to a neutral, upright neck posture. We employed principal component analysis to calculate principal components and the centroid of each vertebral body in MATLAB R2021b (MathWorks, USA) platform. Then, we rotated each vertebral body with regard to its own centroid and matched its first three principal components with the global coordinates of the model. Furthermore, we rotated and translated C2, C3, C4, C5, C6, and C7 vertebrae to match the frontal and sagittal planes of the C1 vertebra.

#### Mesh generation

**Fig. 2:**
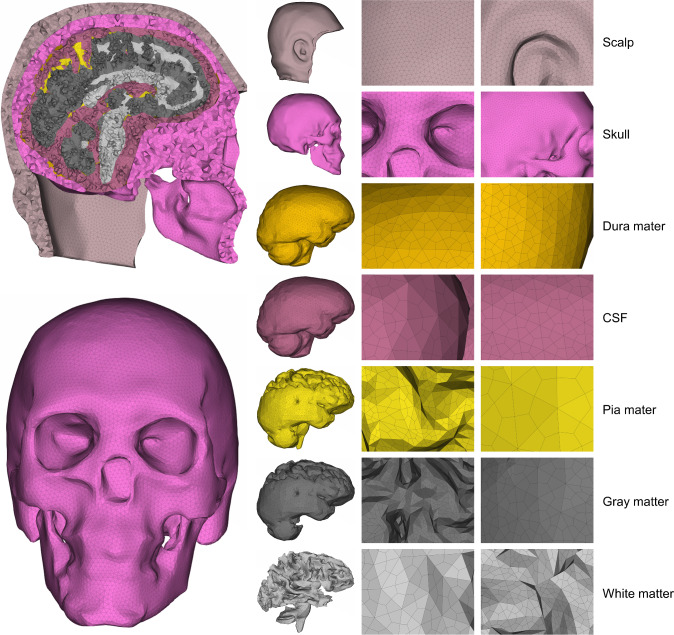
An exploded view of the head finite element model displays the schematics of the mesh structures of scalp, skull, dura mater, CSF, pia mater, brain’s gray matter, and brain’s white matter

**Fig. 3:**
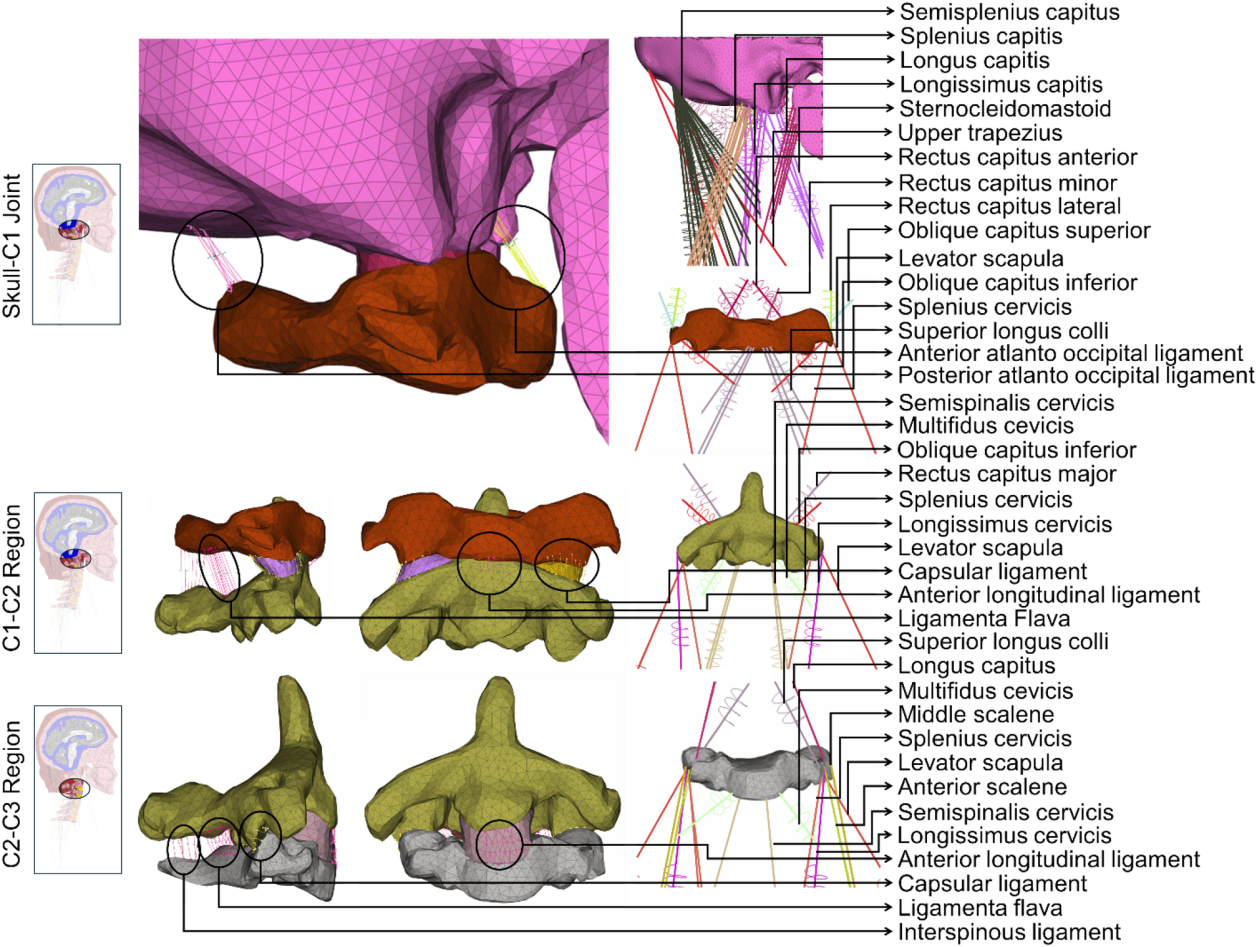
An exploded view of the upper neck finite element model to display the schematics of the mesh structures of the skull, C1, C2, C3, C2-C3 disk, cervical muscles, and ligaments from the skull to the C3. The included cervical muscles are obliquus capitis superior, superior longus colli, rectus capitus major, rectus capitus minor, longus capitis, rectus capitis ant, rectus capitis lat, anterior scalene, middle scalene, posterior scalene, sternocleidomastoid, longissimus capitis, longissimus cervicis, multifidus cervicis, semisplenius capitus, semispinalis cervicis, splenius capitis, splenius cervicis, levator scapula, oblique capitus inferior, and trapezius. The included ligaments are anterior longitudinal ligament, posterior longitudinal ligament, ligamentum flavum, capsular ligament, interspinous ligaments, tectorial membrane, anterior and posterior atlanto-occipital ligaments, anterior and posterior atlanto-axial ligaments, apical ligament, alars ligament, transverse ligament, and cruciate ligament of atlas

**Fig. 4:**
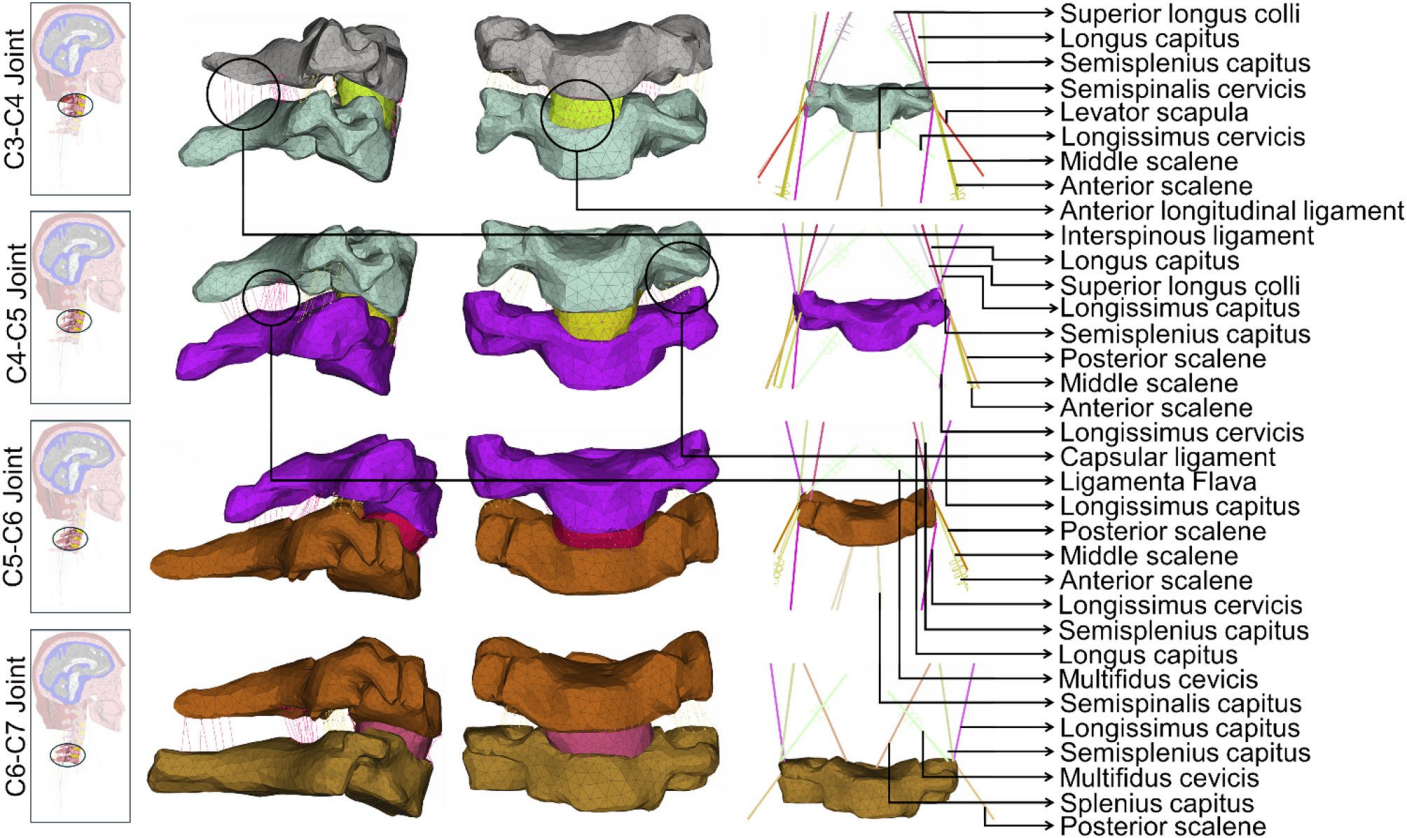
An exploded view of the middle and lower neck finite element model to display the schematics of the mesh structures of C3, C4, C5, C6, and C7 vertebrae, C3-C4, C4-C5, C5-C6, and C6-C7 disks, cervical muscles, and ligaments from C3 to C7. The included cervical muscles are obliquus capitis superior, superior longus colli, rectus capitus major, rectus capitus minor, longus capitis, rectus capitis ant, rectus capitis lat, anterior scalene, middle scalene, posterior scalene, sternocleidomastoid, longissimus capitis, longissimus cervicis, multifidus cervicis, semisplenius capitus, semispinalis cervicis, splenius capitis, splenius cervicis, levator scapula, oblique capitus inferior, and trapezius. The included ligaments are the anterior longitudinal ligament, posterior longitudinal ligament, ligamentum flavum, capsular ligament, interspinous ligaments, tectorial membrane, anterior and posterior atlanto-occipital ligaments, anterior and posterior atlanto-axial ligaments, apical ligament, alars ligament, transverse ligament, and cruciate ligament of atlas

We used the ANSA platform to generate highly detailed, fine meshes for all structures within the head-neck region. The mesh quality of the model was controlled by setting the threshold values of 4 for aspect ratio, 45 for skewness, 20 for warping, and 140 for the maximum angle during the mesh generation process. The resulting meshed model comprised over 1.46 million elements, and the mesh size, element type, and number of elements of individual structures are provided in Table 2. Their detailed mesh schematics are provided in Fig. 2 (head and brain components), Fig. 3 (skull to C3), and Fig. 4 (C3–C7). We modeled the brain’s gray and white matter and CSF as 3D hexahedral meshing, pia and dura mater as 2D-Quad shell, and the rest of the head-neck structures as first-order 3D tetrahedral meshing (Figs. 2, 3 and 4). In the neck region, we included 42 muscles on both the left and right sides and 14 ligaments (Figs. 3 and 4). The origin and insertion points of both muscles and ligaments are, respectively, provided in Supplementary materials Tables S1 and S2. We modeled the ligaments as linear springs with 486 beam elements, and their stiffness was adopted from the literature (Zhang et al. 2005). Similarly, neck muscles were also modeled as beam elements, with a total of 132 elements. The origin and insertion points of these muscles and ligaments were adopted from Gray’s Anatomy book (Standring 2021).

**Table 2:** The mesh structure details of individual head-neck components

Model part	Number of elements	Average mesh size (mm)	Jacobian	Element type	Aspect ratio < 3
Scalp	267,720	3.37 ± 0.98	> 0.8	3D Tetrahedral	100%
Skull	230,473	3.37 ± 1.29	> 0.8	3D Tetrahedral	99%
Dura mater	36,804	1.67 ± 1.20	> 0.8	2-D Quad shell	80%
Pia mater	13,536	2.43 ± 1.14	> 0.8	2-D Quad shell	84%
CSF	138,316	1.68 ± 1.20	> 0.4	3D Hexahedral	88%
Gray matter	360,240	1.31 ± 1.21	> 0.4	3D Hexahedral	85%
White matter	236,188	1.25 ± 0.99	> 0.4	3D Hexahedral	94%
C1 vertebrae	8540	2.55 ± 0.69	> 0.8	3D Tetrahedral	99%
C2 vertebrae	11,378	2.48 ± 0.73	> 0.8	3D Tetrahedral	99%
C3 vertebrae	7860	2.30 ± 0.76	> 0.8	3D Tetrahedral	99%
C4 vertebrae	8344	2.34 ± 0.69	> 0.8	3D Tetrahedral	99%
C5 vertebrae	8055	2.41 ± 0.74	> 0.8	3D Tetrahedral	99%
C6 vertebrae	10,816	2.42 ± 0.74	> 0.8	3D Tetrahedral	99%
C7 vertebrae	10,658	2.48 ± 0.71	> 0.8	3D Tetrahedral	99%
C0-C1 articulations	1418	2.90 ± 0.59	> 0.8	3D Tetrahedral	99%
C1-C2 articulations	814	2.62 ± 0.50	> 0.8	3D Tetrahedral	99%
C2-C3 disk	1049	2.64 ± 0.53	> 0.8	3D Tetrahedral	99%
C3-C4 disk	861	2.49 ± 0.62	> 0.8	3D Tetrahedral	99%
C4-C5 disk	664	2.65 ± 0.69	> 0.8	3D Tetrahedral	99%
C5-C6 disk	667	2.74 ± 0.64	> 0.8	3D Tetrahedral	99%
C6-C7 disk	770	2.81 ± 0.79	> 0.8	3D Tetrahedral	99%
Muscles	132	–	–	1-D beam	–
Ligaments	486	–	–	1-D beam	–

As shown in Table 2, the mesh sizes of most structures were consistent with previous studies that reported mesh sizes of their models. For instance, some studies have reported mesh sizes around 3 mm for both cervical spines and disks (Meyer et al. 2004; Sun et al. 2024). The average mesh sizes of our model, ranging from 1.25 to 3.37 mm for head structures and 2.30–2.90 mm for cervical vertebrae and disks, fall within the range reported in the literature data (see Supplementary Table S3 for detailed comparison between our model and other models in the literature). Additionally, the majority of head-neck FE models did not report mesh sizes, except for the brain. Our brain mesh contains approximately 600,000 hexahedral elements with an average size of 1.3 mm, which is comparable to existing models that range from 50,000 to 1,000,000 elements and average element sizes between 1.5 ~ 7 mm (Supplementary Table S3).

#### Tetrahedral–to–hexahedral mesh conversion for the brain and CSF

**Fig. 5:**
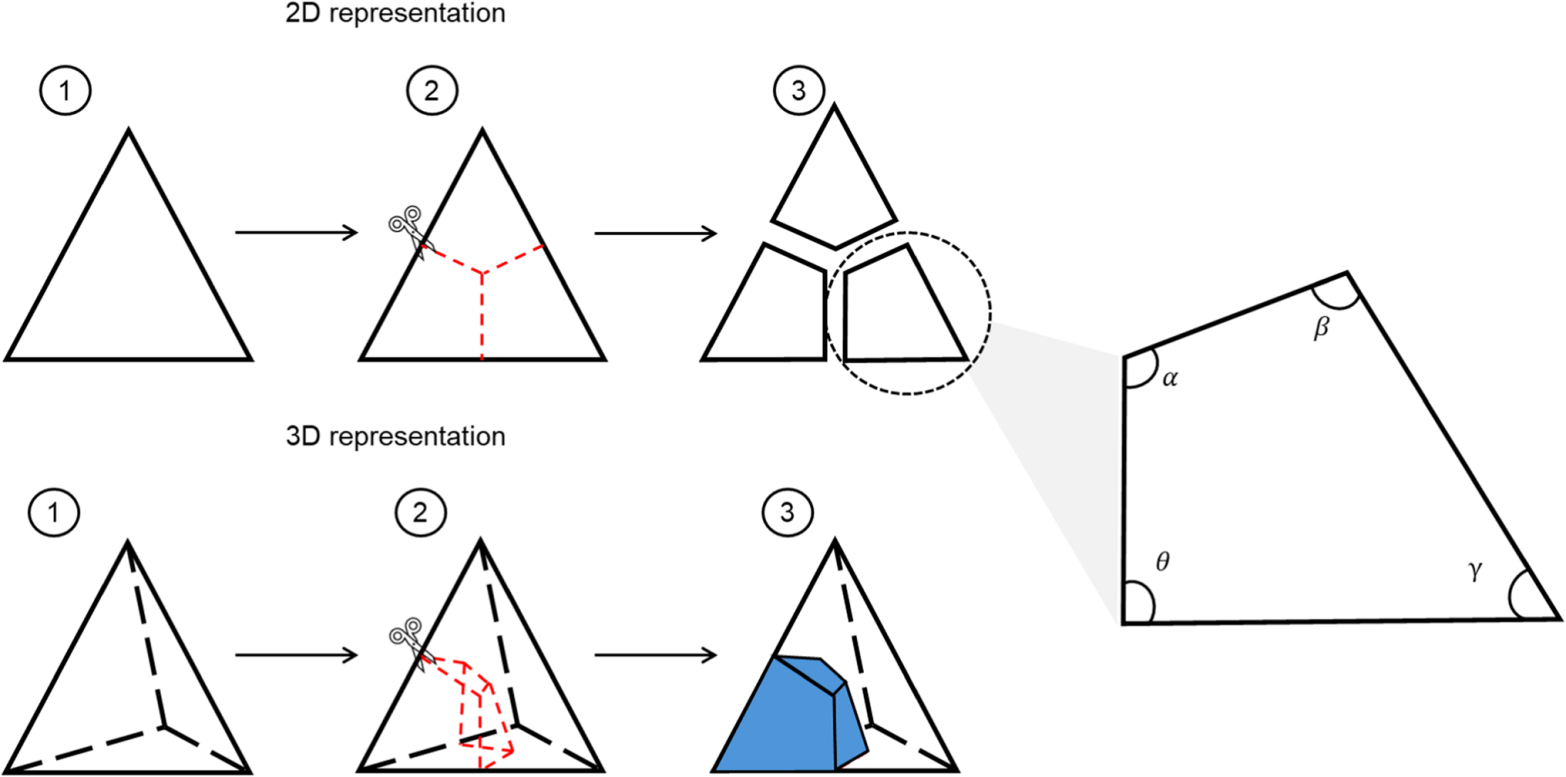
Schematic illustration of the tetrahedral to hexahedral (tet-to-hex) conversion process used for brain and CSF meshing. The top row shows a 2D representation of the method: (1) original tetrahedral cross-section; (2) subdivision using midpoints of edges and faces (highlighted in red); (3) resulting 2D hexahedral-like elements. The bottom row shows the corresponding 3D implementation: (1) original tetrahedral element; (2) edge and face subdivision (red lines); (3) resulting 3D hexahedral elements

To model the brain and CSF with a finer hexahedral mesh structure (Fig. 2), we adopted a custom two-step meshing approach (Fig. 5). First, an unstructured tetrahedral mesh was generated to closely follow the intricate geometry of the gyri, sulci, and narrow CSF pathways. Next, we applied a tetrahedral–to–hexahedral (tet-to-hex) conversion method that replaced each tetrahedral element with four hexahedral elements. This conversion connected the midpoints of tetrahedral edges to the adjacent face midpoints and the element centroid, then cut the whole tetrahedral element into four 8-noded hexahedral elements. During this conversion, each triangular face of the tetrahedral mesh is split into three quadrilateral faces. In an ideal conversion scenario, the triangle face that has equal angles of 60°, the resulting quadrilateral face should have angles of 120°, 90°, 60°, and 90°. However, when the triangle deviates from its ideal shape, the resulting quadrilateral face may incur a distorted shape. In order to maintain the element quality and avoid numerical issues during the simulations, a constraint was employed to enforce all hexahedral angles to be within 140°. After the meshing process, we evaluated the resulting hexahedral mesh using standard mesh quality criteria (Fig. 1). We also checked the Jacobian of the final brain mesh and found all elements to be greater than 0.4 (Davis et al. 2015; Li 2021; Lyu et al. 2022). Fewer than 1000 elements out of nearly 600,000 required manual adjustment using ANSA’s mesh improvement tools. This minimal adjustment did not notably impact computational efficiency or introduce significant bias.

Additionally, the hexahedral meshing could suffer from hourglass modes during dynamic simulation. To prevent hourglass modes, we used appropriate element formulations. For the brain’s gray and white matter regions, fully integrated hexahedral elements (ELFORM = − 2 in LS-DYNA) were used, which inherently eliminate hourglass deformation. Only the CSF domain used reduced integration hexahedral elements (ELFORM = 1) to improve computational speed. For these, hourglass control (IHQ = 6) was applied to suppress artificial energy growth and element distortion (Belytschko and Bindeman 1993). Tetrahedral elements, which were used in other tissues such as the skull and scalp, do not exhibit hourglass behavior, so no hourglass control was necessary.

#### Defining material properties and model assembly

The material properties and governing equations of all head-neck structures included in our model are provided in Table 3. Briefly, we modeled skull, pia mater, dura mater, and vertebrae as linear elastic material (Salimi Jazi et al. 2016; Tse et al. 2015; Tuchtan et al. 2020), scalp as a linear viscoelastic material model (Mao et al. 2013), CSF as nearly incompressible one-term hyper-elastic material (Cotton et al. 2016), intervertebral disks as a hyper-viscoelastic material model (Castro et al. 2014), and brain (gray and white matter) as a hyper-viscoelastic material (Bennion et al. 2022; Ramzanpour et al. 2020). Previous literature (Budday et al. 2015) has shown that white matter is approximately 40% stiffer than gray matter, therefore we modeled the gray and white matters accordingly. We selected brain material properties from the study by (Menichetti et al. 2020) wherein they conducted indentation tests on human cadaver brains with strains up to 35% at a strain rate of 10/s—conditions relevant to TBI cases. As the scalp shows load-rate-dependent mechanical characteristic (Trotta and Annaidh 2019) and has very low stiffness, it is expected to rupture under high-impact loads. Therefore, we applied the erosion model available in the LS-DYNA (Livermore Software Technology Corporation, USA) platform to identify and remove ruptured tissues if the strain of any scalp element exceeded 60%, as found in a previous study on the human cadaver (Jussila et al. 2005). Moreover, we modeled the muscles using the Hill-type muscle model that calculates the total force of a muscle by summing the active force from its active contractile element and the passive force from its elastic component. The active contractile force was a function of time-varying muscle activation, muscle length, and contraction velocity, while the passive force depended on the stretch of the muscle beyond its resting length (Winters 1990). Muscle volume and physiological cross-section area (PCSA) were taken from the literature (Panzer et al. 2011). Muscle resting length was calculated as muscle volume divided by its’ PCSA. The maximum velocity of a muscle was set to 10 times its resting length. The maximum stress of a muscle was set to 0.3 MPa (Winters 1990).

**Table 3:** Material properties and constitutive equations of each head-neck structure included in our model

Segment	Material model	Material constants	Constitutive equation
Scalp (Mao et al. 2013)	Linear viscoelastic	$G_{0} = 1.70 \;{\text{MPa}}, G_{\infty } = 0.68\; {\text{MPa}}$ $K = 20 \;{\text{MPa}}$ $\beta = 0.00003$ $\rho = 1100 \;{\text{kg/m}}^{{3}}$	$G\left( t \right) = G_{\infty } + \left( {G_{0} - G_{\infty } } \right)e^{{ - {\upbeta }t}}$
Skull(Salimi Jazi et al. 2016)	Linear elastic	$E = 15.00 \;{\text{GPa}}$ $\rho = 1800 \;{\text{kg/m}}^{{3}}$ $v = 0.21$	$\sigma = E\varepsilon$
Dura mater (Tuchtan et al. 2020)	Linear elastic	$E = 5.00 \;{\text{MPa}}$ $\rho = 1200 \;{\text{kg/m}}^{{3}}$ $v = 0.45$	$\sigma = E\varepsilon$
Pia mater (Tuchtan et al. 2020)	Linear elastic	$E = 2.30 \;{\text{GPa}}$ $\rho = 1000\; {\text{kg/m}}^{{3}}$ $v = 0.45$	$\sigma = E\varepsilon$
Vertebrae (Tse et al. 2015)	Linear elastic	$E = 8.00 \;{\text{GPa}}$ $\rho = 1200 \;{\text{kg/m}}^{{3}}$ $v = 0.22$	$\sigma = E\varepsilon$
CSF(Cotton et al. 2016)	Hyper-elastic	$C_{10} = 0.0112$ $\rho = 1000\; {\text{kg/m}}^{{3}}$ $v = 0.499$	$W\left( {J_{1} ,J_{2} ,J} \right) = \mathop \sum \limits_{p,q = 0}^{n} C_{{{\text{pq}}}} \left( {J_{1} - 3} \right)^{p} \left( {J_{2} - 3} \right)^{q} + W_{{\text{H}}} \left( J \right)$
White matter (Menichetti et al. 2020)	Hyper-viscoelastic	$\mu_{0} = 7.63\; {\text{kPa}} ,\mu_{\infty } = 1.58\; {\text{kPa}}$ $G_{\infty } = 0.21$ $G_{1} = 0.57 , \tau_{1} = 0.02 s$ $G_{2} = 0.22 , \tau_{2} = 0.31 s$ $p = 1060\; {\text{kg/m}}^{{3}}$ $K = 2.19 \;{\text{GPa}}$	$W = \frac{\mu }{2}\left( {I_{1} - 3} \right) + \frac{K}{2}\left( {J - 1} \right)^{2}$ $G\left( t \right) = G_{\infty } + \mathop \sum \limits_{i = 1}^{N} G_{{\text{i}}} e^{{ - t/\tau_{{\text{i}}} }}$
Gray matter (Menichetti et al. 2020)	Hyper-viscoelastic	$\mu_{0} = 5.06 \;{\text{kPa}} ,\mu_{\infty } = 1.48\; {\text{kPa}}$ $G_{\infty } = 0.29$ $G_{1} = 0.50 , \tau_{1} = 0.015 s$ $G_{2} = 0.20 , \tau_{2} = 0.30 s$ $p = 1060 kg/m^{3}$ $K = 2.19 GPa$	$W = \frac{\mu }{2}\left( {I_{1} - 3} \right) + \frac{K}{2}\left( {J - 1} \right)^{2}$ $G\left( t \right) = G_{\infty } + \mathop \sum \limits_{i = 1}^{N} G_{{\text{i}}} e^{{ - t/\tau_{{\text{i}}} }}$
Intervertebral disks (Castro et al. 2014)	Hyper-viscoelastic	$C_{10} = 0.15, C_{01} = 0.03$ $\rho = 1000 kg/m^{3}$ $v = 0.499$ $G_{1} = 1.70, G_{2} = 1.20 ,G_{3} = 2.00$ $\tau_{1} = 11.76, \tau_{2} = 1.10 ,\tau_{3} = 0.13$	$W\left( {J_{1} ,J_{2} ,J} \right) = \mathop \sum \limits_{p,q = 0}^{n} C_{{{\text{pq}}}} \left( {J_{1} - 3} \right)^{p} \left( {J_{2} - 3} \right)^{q} + W_{{\text{H}}} \left( J \right)$ $G\left( t \right) = \mathop \sum \limits_{k = 1}^{n} G_{{\text{k}}} exp^{{ - t/\tau_{{\text{k}}} }}$
Muscles (Panzer et al. 2011; Winters 1990)	Hill-type	PCSA (Panzer et al. 2011) Muscle volume (Panzer et al. 2011)	$F_{{{\text{active}}}} = F_{{{\text{max}}}} \times f_{{{\text{FL}}}} \times f_{{{\text{FV}}}} \times A\left( t \right)$ $F_{{{\text{Passive}}}} = \frac{{F_{{{\text{max}}}} }}{{e^{{K_{{{\text{sh}}}} }} - 1}}\left[ {e^{{\frac{{K_{{{\text{sh}}}} }}{{L_{{{\text{max}}}} }}\left( {\frac{L}{{L_{{{\text{rest}}}} }} - 1} \right)}} - 1} \right] For L > L_{{{\text{rest}}}}$
Ligaments (Zhang et al. 2005)	Linear spring	k (Zhang et al. 2005)	$F = kx$

*E* Elastic modulus, ${\text{G}}_{0}$ Short-term shear modulus, ${\text{G}}_{\infty }$ Long-term shear modulus, *K* Bulk modulus, ${\upbeta }$ Decay constant, ${\uprho }$ Density, ${\text{v}}$ Poisson ratio, ${\uptau }_{{\text{k}}}$ Relaxation time, ${\text{g}}_{{\text{k}}}$ Relaxation coefficient, *W* Strain energy density function, ${\text{C}}_{{{\text{pq}}}}$, ${\upmu }$ Shear modulus, *F*_active_ Active muscle force, *F*_passive_ Passive muscle force, $f_{{{\text{FL}}}}$ Force-length relation of the muscle, $f_{{{\text{FV}}}}$ Force-velocity relation of the muscle, *A*(*t*) Muscle’s activation level versus time relationship, $L_{{{\text{rest}}}}$ The resting length and $K_{{{\text{sh}}}}$ Passive muscle force constant, *k* ligament stiffness

Similar to previous studies (Ghajari et al. 2017; Mao et al. 2013), we implemented tied boundary contact between disks and vertebrae, skull and scalp, skull and dura mater, dura mater and CSF, CSF and pia mater, brain and pia mater and neck-skull articulations. Moreover, the bottom surface of the C7 cervical vertebra was fixed to restrict its’ all degrees of freedom. Additionally, an automatic contact was set up within the entire neck structure to prevent any self-penetration in extreme scenarios.

### Experimental Data for head-neck FE model validation

**Fig. 6:**
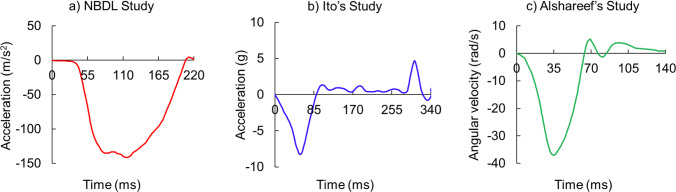
Experimental impact scenarios that were numerically replicated in this study: **a** linear acceleration profile of NBDL study (Ewing and Thomas 1972) with respect to T1 vertebral rotation (Thunnissen et al. 1995), and **b** linear acceleration profile for Ito’s (Ito et al. 2005) cervical vertebrae study, and **c** Angular velocity profile for Alshareef‘s (Alshareef et al. 2018) sonomicrometry study. The run time for finite element simulations of NBDL, Ito’s, and Alshareef’s studies were, respectively, 220 ms, 350 ms, and 150 ms

Three head impact experiments (Alshareef et al. 2018; Ito et al. 2005; Thunnissen et al. 1995) were used to validate the efficacy of our developed head-neck FE model (Fig. 6). We used LS-DYNA explicit solver platform installed in the high-performance computing center (two AMD EPYC 7702 CPUs and 500 GB memory) to solve all impact simulations. A simulation of 220 ms (NBDL study) took about 36 h to complete.

#### NBDL study

The first experimental dataset was the linear acceleration profile (Fig. 6a) of an in-vivo study conducted by the Naval Biodynamics Laboratory (NBDL) in 1972 (Ewing and Thomas 1972). Later, (Thunnissen et al. 1995) corrected NBDL’s linear acceleration profile for *T*1 vertebral rotation in 1995. We applied one of those corrected acceleration profiles (Thunnissen et al. 1995) (Fig. 6a) to our whole head-neck model. As the subject’s neck muscles were pre-tensed in the NBDL experiment, we modeled the flexor muscles with 10% activation and the extensor muscles with 80% activation. These activation levels were determined via a trial-and-error process that provided experimental neck kinematics. Additionally, this study was also used to validate the biomechanical fidelity of the cervical spine, as we estimated the neck kinematics by summing the motions of C0-C1 to C6-C7.

#### Ito’s study

Our third scenario involved data from (Ito et al. 2005) a cervical vertebrae study to validate how our modeled neck responds to linear impact in comparison with the experiment. To do so, we applied their linear acceleration profile to the C7 vertebrae (Fig. 6b), where they subjected the neck to an 8 g horizontal acceleration and measured disk strain. To replicate their muscle force simulation, which employed 4.0 N/mm springs for anterior and lateral springs and 8.0 N/mm for posterior springs, we adjusted our model with an 80% activation level for neck extensor muscles and a 40% activation level for neck flexor muscles. These activation levels were determined through a trial-and-error process such that the intervertebral disk strain of our model matched those reported in Ito’s study.

#### Alshareef’s study

Our third experiment drew from (Alshareef et al. 2018)’s head-neck cadaver study wherein they measured brain displacement using Sonomicrometry crystals in response to four dynamic pure rotational pulses (20 and 40 rad/s for 30 and 60 m/s) in all three directions, however they reported the brain deformation and kinematics in response to pulses in the coronal direction. Therefore, in this study, we simulated the coronal pulse of 40 rad/s and 60 ms duration in pure lateral direction (Y-direction of the Alshareef’s study) and measured corresponding brain displacement and brain strain to validate the mechanical response of our modeled brain (Fig. 6c). As neck muscles are not active in cadavers, we modeled our muscles with 0% activation for this simulation. Furthermore, we scaled our whole head-neck model by a factor of 0.92 to match their brain weight of 1.265 kg (Alshareef et al. 2018). Additionally, (Alshareef et al. 2018)’s study reported only the brain displacement data from three out of 24 crystals or receivers (receivers 9, 16, and 3) that they placed around the brain. Therefore, we measured brain displacement in those three receivers in the axial plane, which are located in the parietal region of the brain. It should be noted that the locations of some receivers were approximated because the structural shapes of our scaled model were slightly different than (Alshareef et al. 2018)’s head-neck cadaver. Additionally, we measured the maximum principal strain (MPS) of the whole brain as well as at all 24 receiver locations. The peak MPS values of our simulation were compared against the study by (Wu et al. 2019), who simulated the same dynamic pulse from (Alshareef et al. 2018)’s Sonomicrometry study to validate the mechanical responses of their solid brain and axon-based brain models.

### Post-processing and statistical analysis

We used META (BETA CAE Systems SA, Greece) software for post-processing and retrieving numerical solutions of all three impact simulations. We compared model-predicted neck flexion angle data and their peak time with the experimental neck flexion data of the NBDL study (Thunnissen et al. 1995). To compare with the actual experimental results of Ito’s study (Ito et al. 2005), we calculated cervical disk strain at every disk level. In addition, we conducted an evaluation of brain deformation at specific points highlighted in Alshareef’s study (Alshareef et al. 2018) to validate our own brain deformation findings. Pearson correlation analysis was employed for pairwise comparison between all numerical and experimental data patterns. For this purpose, we digitized Alshareef’s experimental brain deformation results (Alshareef et al. 2018) NBDL’s experimental neck flexion angle plot (Thunnissen et al. 1995) in the MATLAB platform. For the same digitized points, we retrieved neck positional information in the META platform. The neck flexion angle was the angle between the vertical line and the line joining the anterior-most point of the foramen magnum and the anterior-inferior corner-most point of C7 in the mid-sagittal plane. Furthermore, it's important to note that each of these experimental studies utilized distinct coordinate systems. To ensure a precise and meaningful comparison between our numerical findings and their experimental data, we transformed our results to align with the coordinate systems employed in each respective study.

## Results

### 3D head-neck geometry

The comparison between our model’s head-neck geometry data against experimentally measured values reported in the literature is provided in Table 4. The head geometry data exhibited that both the skull thickness and brain volume of our model were within one standard deviation (SD) of the values reported in previous literature (Filipek et al. 1994; Hagemann et al. 2008) (Table 4). On the other hand, the model's CSF volume was found to be larger than the literature data (Grant et al. 1987)—about 29.52% greater than the maximum reported value. The maximum scalp thickness value was also observed to be 25.70% higher than the literature data (Babiloni et al. 1997).

**Table 4:** Comparison between the geometrical parameters of head and neck structures and previously-reported experimental values. *F*3, *F*4, *T*3, *T*4, *P*3, *P*4 indicate skull measurement locations at frontal left, frontal right, temporal left, temporal right, parietal left, and right parietal skulls, respectively

Parameter	References	Model
**Scalp thickness (mm)**	Min: 3.0–Max: 14.2 (Babiloni et al. 1997)	Min: 3.14–Max: 17.85
**Skull thickness (mm)**	(Hagemann et al. 2008)	
*F*3	5.95 ± 1.40	6.54 ± 1.43
*F*4	6.16 ± 1.32	5.51 ± 0.61
*T*3	3.31 ± 0.80	3.93 ± 0.19
*T*4	3.46 ± 0.78	3.15 ± 0.41
*P*3	6.36 ± 1.30	6.84 ± 0.55
*P*4	6.09 ± 1.25	5.76 ± 0.81
**Brain volume (cm**^**3**^**)**	1173.33–1625.66 (Filipek et al. 1994)	1322.32
**Cranial CSF volume (cm**^**3**^**)**	57.1–286.5 (Grant et al. 1987)	371.10
**Vertebral height (mm)**	(Vasavada et al. 2008)	
C3	13.5 ± 0.7	11.68 ± 0.56
C4	12.6 ± 0.8	13.47 ± 0.46
C5	12.2 ± 0.6	12.34 ± 0.65
C6	12.0 ± 0.7	11.93 ± 1.70
C7	13.0 ± 1.0	12.41 ± 2.23
**Vertebral Depth (mm)**	(Vasavada et al. 2008)	
C3	14.2 ± 1.6	15.47 ± 0.56
C4	14.8 ± 1.2	14.53 ± 1.47
C5	15.2 ± 1.1	16.28 ± 2.36
C6	15.6 ± 1.0	19.42 ± 1.41
C7	15.5 ± 1.3	18.73 ± 0.67
**Spinous process length (mm)**	(Vasavada et al. 2008)	
C2	20.9 ± 3.5	20.18
C3	18.8 ± 2.4	19.64
C4	19.3 ± 3.0	26.76
C5	22.4 ± 3.4	25.74
C6	27.7 ± 4.6	27.57
C7	34.9 ± 3.8	34.24
**Vertebral body to spinous process length (mm)**	(Vasavada et al. 2008)	
C2	35.8 ± 3.6	38.09
C3	30.4 ± 2.0	33.02
C4	30.1 ± 2.8	33.85
C5	32.5 ± 2.9	33.37
C6	39.7 ± 5.1	36.23
C7	46.9 ± 3.4	48.23
**Dimensionless Intervertebral Disk Height**	(Frobin et al. 2002)	
C2/C3	0.35 ± 0.07	0.303
C3/C4	0.38 ± 0.08	0.366
C4/C5	0.39 ± 0.06	0.375
C5/C6	0.38 ± 0.04	0.295
C6/C7	0.36 ± 0.06	0.323

### FE simulation results

#### NBDL study

**Fig. 7:**
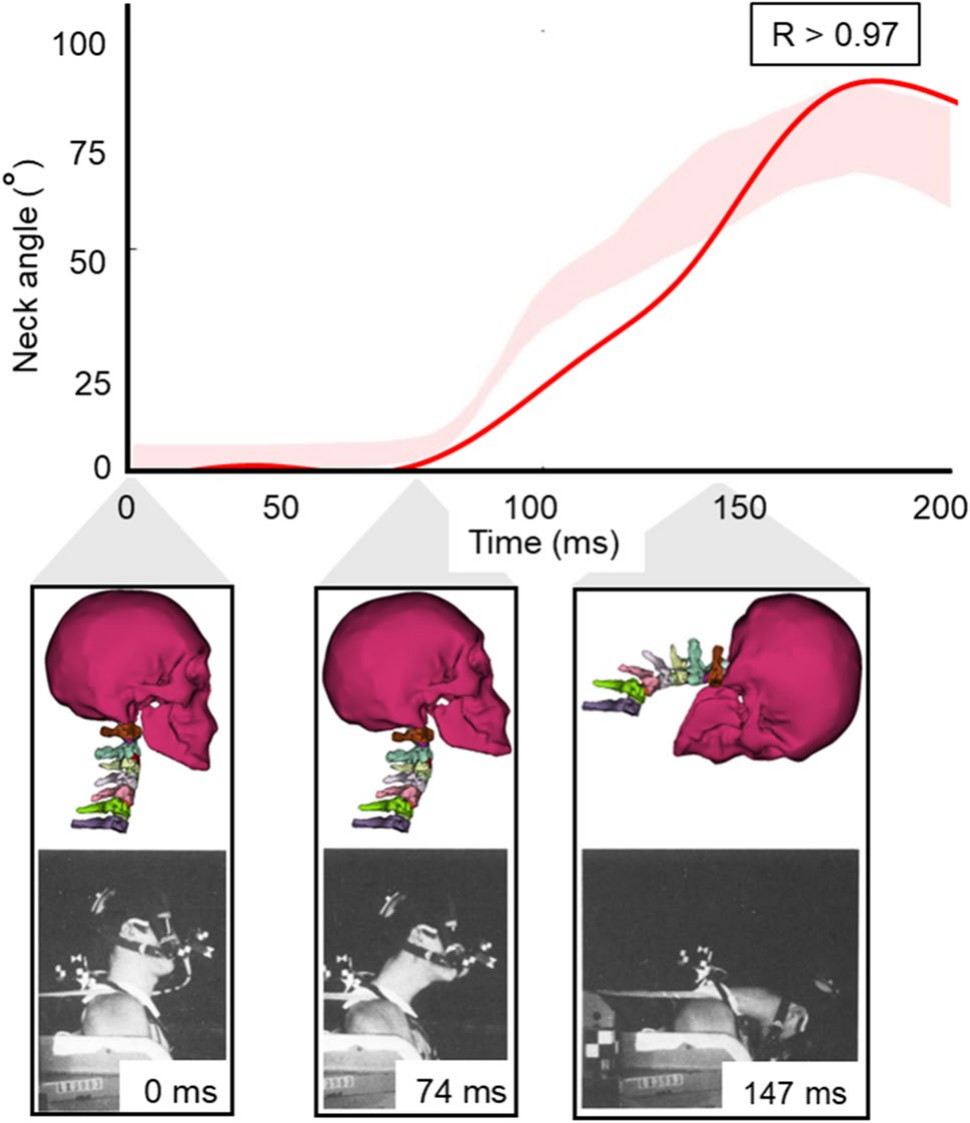
Comparison between model-predicted and NBDL-experimental neck flexion angles for the complete simulation duration (top) and a visual comparison between model-prediction (top) and experimental head-neck kinematic responses (bottom) at three different time instances (Thunnissen et al. 1995). The shaded area represents the experimental range of neck angles, while the solid line indicates the corresponding simulation results

Our model’s neck kinematic responses strongly matched to the NBDL's acceleration profile (Fig. 7). Pearson’s correlation analysis evinced a strong positive correlation (*r* > 0.97) between experimental and numerical head-neck kinematic patterns. The model showed a neck flexion angle of 36.45° ± 34.14°, with a peak of 88.31°, whereas the NBDL study reported 11 subjects' neck flexion angles, ranging between a mean angle of 39.69° ~ 30.72° and a maximum angle of 67.20° ~ 87.50° (Thunnissen et al. 1995). Though a small number of discrepancies were found for the maximum neck angle (0.92% larger) and its peak time (5 ms delay), the visual comparisons of kinematic behavior at three time instances revealed analogous head-neck responses between model predictions and experimental simulations (Fig. 7).

#### Ito’s study

**Fig. 8:**
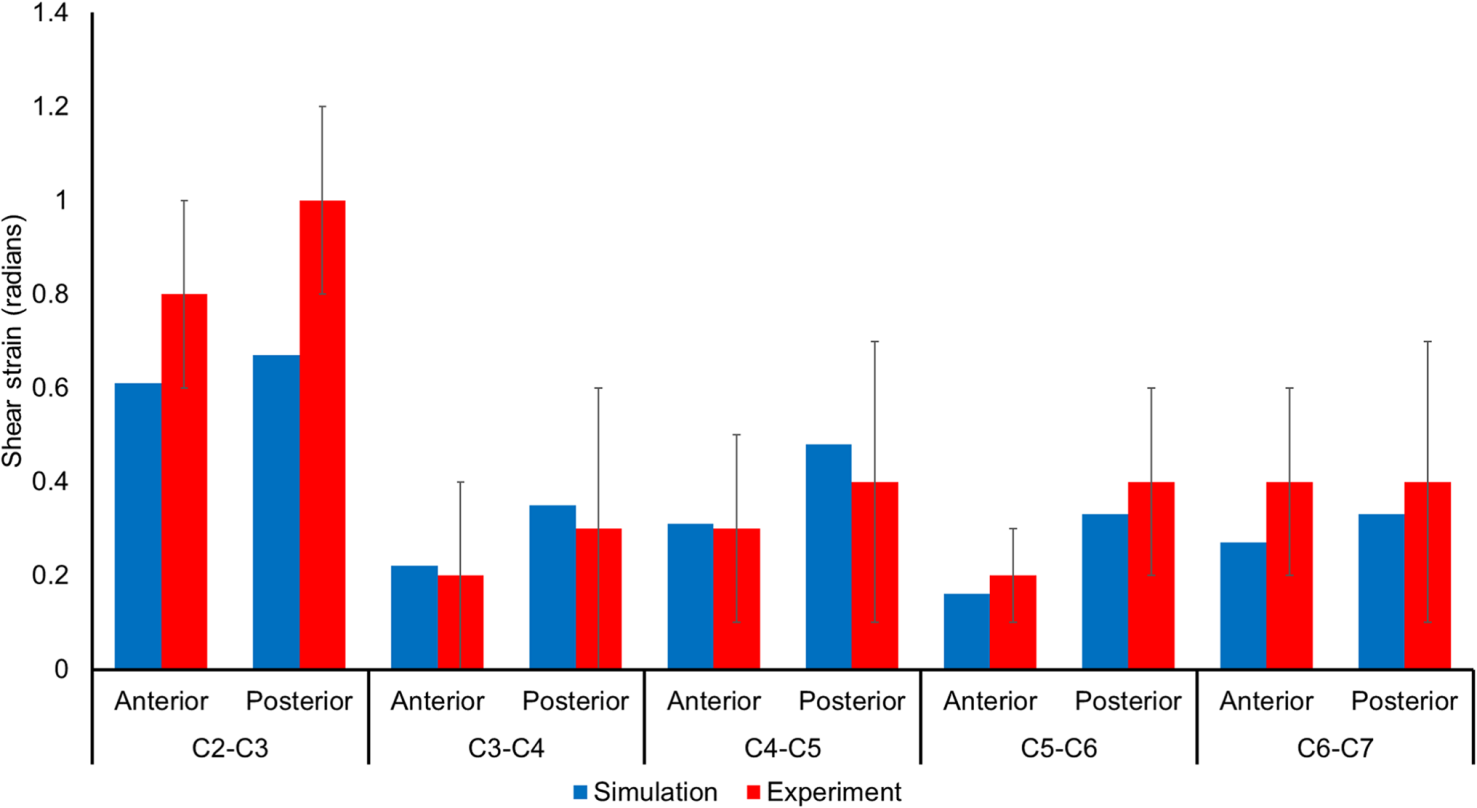
Comparison between simulated and experimental (mean ± SD) peak shear strain (Ito et al. 2005) in an 8 g frontal impact Scenario

Our in-silico replication of Ito’s experimental study conditions (Ito et al. 2005) yielded peak shear strain values that were found to lie within one standard deviation of Ito’s experimental shear strain values for both anterior and posterior regions of individual intravertebral disks except the posterior region of the C2–C3 disk (Fig. 8) wherein the peak shear strain was more than one standard deviation but within the two standard deviations from its experimental counterpart. A notable difference in the peak shear strain values between the experimental and simulation was also found in the C2–C3 disk.

#### Alshareef’s study

**Fig. 9:**
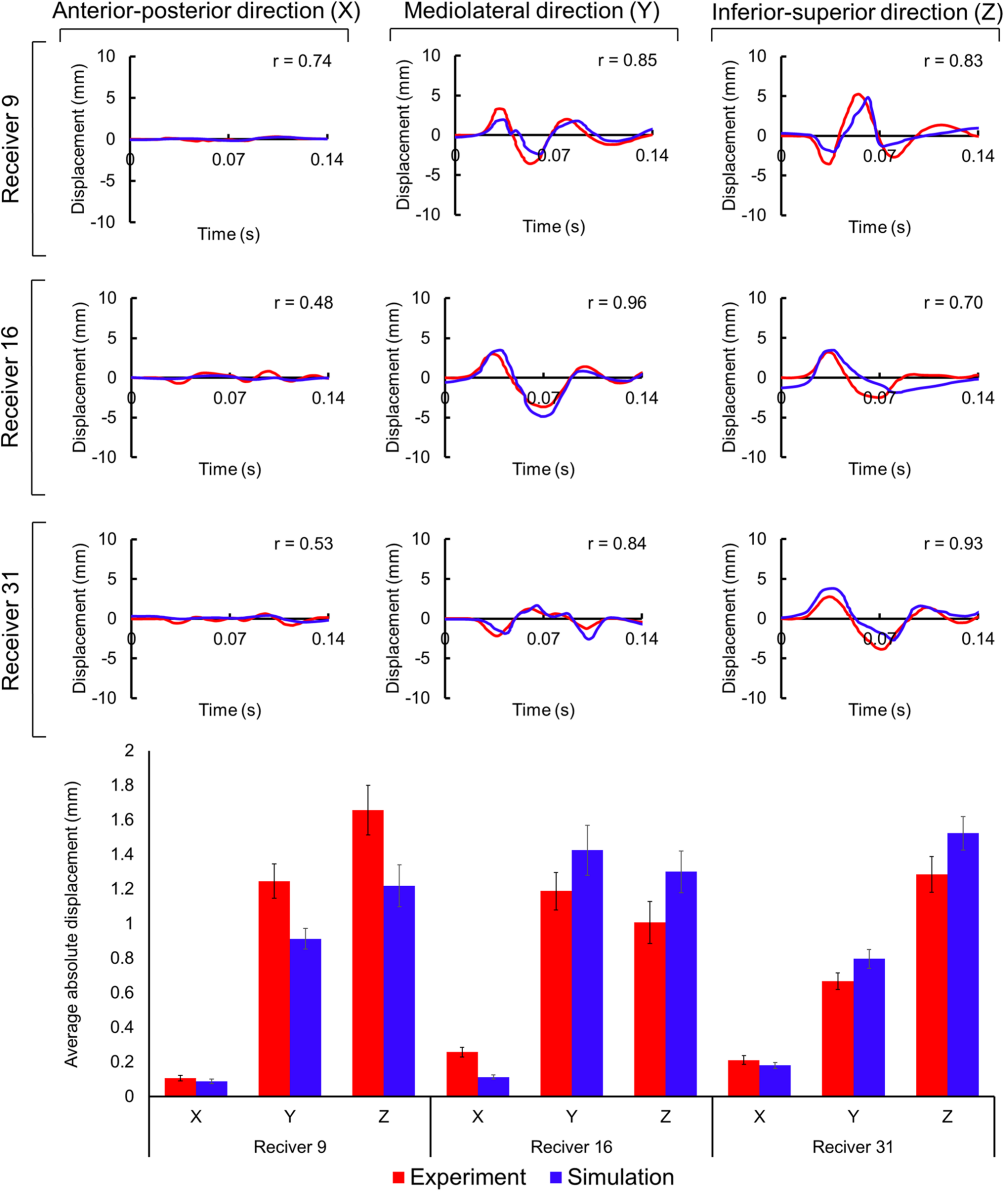
Comparative analysis of brain displacement between simulated and experimental results (Alshareef et al. 2018) in *X*, *Y*, and *Z* directions across three receiver locations. Corresponding correlation coefficients (*r*) are included for each case. Comparison of simulated average absolute displacement values with experimental counterparts (mean ± SD)

**Fig. 10:**
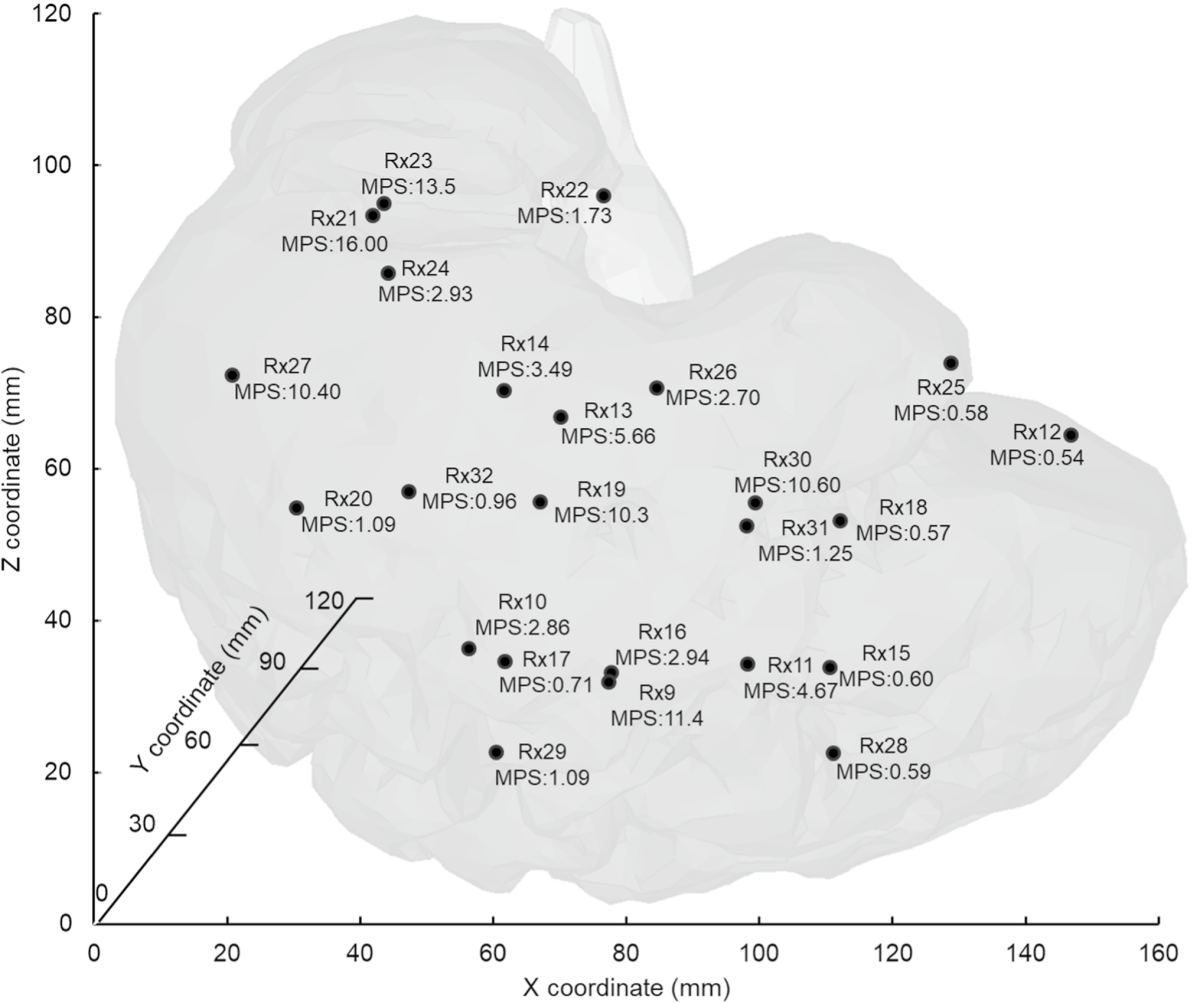
A sagittal view of the 3D brain model with the approximate locations of all 24 receivers. The peak maximum principal strain (MPS) values of all receivers are in percentage (%). The peak MPS for the overall brain was 40.20%

Our brain displacement outcomes in the *Y* (medio-lateral) and *Z* (superior-inferior) directions at all three locations demonstrated high correlation (*r* = 0.70 ~ 0.96) with the experimental findings (Alshareef et al. 2018), as depicted in Fig. 9. In contrast, our displacement results in the *X* (anterior-posterior) direction displayed a relatively weaker level of correlation (*r* = 0.48 ~ 0.74). In this scenario, as the displacements fluctuate around zero, computing a simple average of the data would yield a value close to zero, irrespective of the amplitude of these oscillations. Consequently, we opted to determine the average of the absolute displacements. This approach enables us to effectively compare the displacement magnitudes between our numerical results and the experimental data (Fig. 9). The results also revealed disparities of 17.64%, 26.63%, and 26.49% between our numerical average absolute displacements and their experimental counterparts in the *x*, *y*, and *z* directions for receiver 9 location. In the case of receiver 16 locations, these differences amounted to 56.34%, 19.83%, and 29.05% for the *X*, *Y*, and *Z* directions. For the receiver 31 location, we observed differences of 14.99%, 19.54%, and 18.60% in the *X*, *Y*, and *Z* directions, respectively. Furthermore, Fig. 10 shows the peak MPS values at all 24 receiver locations from the simulation of Alshareef’s study. The highest strain was found at receiver 21, located in the occipital region, with a peak of 16.00%. On the other hand, areas in the frontal and parietal regions had lower peak values. The peak MPS of the whole brain was observed to be 40.20%, which was similar to those reported by (Wu et al. 2019) study.

## Discussion

In this study, we developed a biofidelic head-neck FE model from MRI datasets and validated its’ biomechanical responses against three experimental datasets (Alshareef et al. 2018; Ito et al. 2005; Thunnissen et al. 1995). The numerical solutions revealed that our developed model was capable of simulating the experimental impact scenarios and predicted the biomechanical response of model structures (e.g., brain stress–strain tensor values) significantly well.

### Model geometry validation

The results showed that most geometrical measures were within the 3 $\sigma$ range of previously reported values (Table 4). Only scalp thickness and CSF volume of our model were somewhat larger than their corresponding values in the literature (Babiloni et al. 1997; Grant et al. 1987). In order to verify that they were not erroneously segmented in the T2 MRI sequence, we also imaged and segmented T-1 weighted images of the same subject and found the same scalp thickness and CSF volume. Additionally, previous studies reported that skin thickness is correlated with body weight (Pedersen et al. 1995). As our study participant was a 92nd percentile male by weight, thus the subject had a higher-than-average scalp thickness (Paquette 2009). Additionally, we defined CSF volume as the space between the skull and brain, which includes dura and pia maters, intracranial blood vessels, meninges, etc. As we did not model other structures except dura and pia maters between the skull and brain, we conjecture that the inclusion of their spaces as CSF led to a larger CSF volume. The scalp was approximately 26% thicker, and the cranial CSF volume was about 30% larger than the upper bound values reported in population data. These anatomical differences might have influenced the mechanical response of the model. For example, the thicker scalp might have absorbed more energy during impact, and the larger CSF volume might have increased the cushioning effect around the brain.

Six out of 22 cervical vertebral measures (C3 height, C6 and C7 depth, C4 spinous process length, and C3 and C4 vertebral body to spinous process length) (Vasavada et al. 2008) and the C5/C6 disk height (Frobin et al. 2002) were found to marginally exceed their normal range, as reported in a previous study (Vasavada et al. 2008). A few previous studies have also reported such geometrical discrepancies in the neck region (Barker and Cronin 2021; Liang et al. 2022; Vasavada et al. 2008). Such as, Liang et al. (Liang et al. 2022) observed larger C2 and C3 spinous process lengths, and (Barker and Cronin 2021) found higher posterior disk height than the reported literature data. This leads us to believe that, regardless of the imaging technique and the study subject’s anthropometric distribution, voxel-based intensity standardization methods (Wahid et al. 2021) should be implemented to enhance soft- and hard-tissue contrasts in the complex neck region. The lack of such an image processing method might have led to slight discrepancies in those vertebral measures, even though we rigorously segment and reconstruct the 3D geometry of each neck structure.

### The model’s biomechanical performance

When a mechanical impact is introduced to the head and neck system, the resultant movements (and/or stress) transfer from one tissue to another and cause deformations in every head-neck structure. Therefore, it is crucial to ensure that our model is able to accurately reproduce these events and assess the mechanical behavior of all head-neck structures. For this purpose, we simulated three experimental studies: (1) NBDL study (Thunnissen et al. 1995) (high linear acceleration impacts that are commonly seen in automotive and aviation accidents), (2) Ito’s study (Ito et al. 2005) (simulated frontal impact to assess the biomechanical response of the cervical spine), and (3) Alshareef’s sonomicrometry study (Alshareef et al. 2018) (brain displacement and brain strain in response to angular impact commonly seen in American football).

#### NBDL study: head-neck kinematics validation

Around 80–90 ms in the NBDL study, the model slightly overpredicts the neck flexion angle relative to the range observed in the experimental data. This discrepancy may stem from the use of static muscle activation levels in the current simulation, which do not fully capture the rapid neuromuscular responses occurring during this dynamic phase. Incorporating time-dependent or EMG-informed muscle activation profiles in future simulations may improve model accuracy during this period. Additionally, as we incorporated certain geometric and mechanical simplifications (e.g., linear elastic properties of bones and ligaments) in our original model, we expected slight differences between in-vivo results (Thunnissen et al. 1995) and our model predictions. However, the high correlation (*r* > 0.97) between neck flexion angle profiles and the minor difference (5 ms) in their peak time between NBDL experimental and numerical simulations indicate that our model’s head-neck damping characteristics closely approximate those of a living human, which is especially noteworthy since NBDL employed live human subjects rather than cadavers as in other studies (Fig. 7). This highlights the fidelity of our model's neck damping characteristics to that of living humans.

#### Ito’s cervical spine study: intervertebral disk strain

For Ito’s study (Ito et al. 2005), the most significant disparity between our values and the experimental mean was observed for C2-C3 intervertebral disk, especially the posterior region of the disk, where our simulated peak shear strain fell within two standard deviations of the experimental value. Like our study, (Barker and Cronin 2021) have also sought to validate their neck response using Ito's frontal impact experimental results. They observed higher model-predicted peak shear strain values than the experimental results. Particularly, the anterior peak shear strain at C3-C4, C4-C5, and C5-C6 disks and the posterior peak shear strain at C3-C4 and C4-C5 disks were higher than one standard deviation. This discrepancy could be caused by our model’s simplifications in the neck region, such as a simplified representation of ligaments as linear springs. On the other hand, the differences in the peak shear strain can be attributed to the use of an artificial head in the experiment. Although our model's head possesses similar weight and moment of inertia characteristics to the artificial head used by Ito et al., the proximity of the C2-C3 disk to the head could magnify the impact of the artificial head's influence on the results. Furthermore, Ito’s experimental study used constant and equal force springs for muscle activation, differing from our approach of distinct, but constant activation levels for flexors and extensors.

#### Alshareef sonomicrometry study: brain displacement dynamics

We found a high correlation between the brain displacement results in the *Y* (medio-lateral) and *Z* (superior-inferior) directions and a medium level of correlation in the *X* (anterior–posterior) direction (Fig. 9). As Alshareef’s experimental motion occurs in the *Y*–*Z* plane (lateral direction), minor *X*-direction displacements were observed in both experiment and simulation. These displacement values were much smaller (< 1 mm) than the displacements (> 5 mm) in the *Y* or *Z* direction. Consequently, we observed a lower correlation value in the *X*-direction. Additionally, the absolute brain displacement values in the *Y*-direction for Receiver 9 were also found to lie outside one standard deviation of the experimental value. Previous FE study by the same author has also found such discrepancies, where they reported a Correlation and Analysis objective rating system (CORA) score of 0.4–0.6 between their numerical results and Alshareef’s experimental data (Wu et al. 2019). Furthermore, as brain displacement kinematics depends on the brain-fluid-skull relative motion, the lack of fluid–structure interface modeling of brain-CSF and CSF-skull interfaces contributed to the discrepancies in the brain displacement results. The brain strain results suggest that the occipital region, as shown by the highest MPS at receiver 21, could generally be more vulnerable to TBI impacts which are purely in the lateral direction of the coronal plane. Nonetheless, the peak MPS of the whole brain (40.20%) was significantly greater than those observed across all receivers and falls between the values reported by (Wu et al. 2019) for their isotropic solid brain (47%) and embedded axonal brain (33%) models, showing that our model captures strain levels within a realistic range with slight differences that could be attributed to the lack of an embedded anisotropic axonal fibers in our model. Overall, though brain displacement and brain strain results show that our model can capture overall brain deformation well, however adding anisotropic axonal fibers to our brain model could better assess injury risk.

### Study limitations

This study had several limitations. *First*, similar to many previous studies, we modeled the brain gray and white matter as hyper-viscoelastic materials with isotropic properties (Ghajari et al. 2017; Giudice et al. 2021). In order to capture the actual brain anisotropic properties, an axonal brain model should be used. Though there are a few brain axonal tissue models (Chatelin et al. 2011; Li et al. 2021), they, however, lack either neck components or many other head components. It is an active research area in the status quo to couple the brain axonal model with the brain solid model in order to study brain anisotropic responses under various mechanical impacts. *Second*, the model allows the muscles to simulate time-variant complex activation patterns. However, in this study, we determined our muscle activation strategies through a trial-and-error process in the FE platform to match the experimental neck kinematics and intervertebral disk responses, For example, our trial-and-error process yielded muscle activation level of 80% for extensors and 40% for flexors that match Ito’s study simulation (disk strain level) and 80% for flexors and 10% for extensors in the NBDL study simulation (neck kinematics). *Third*, we did not implement voxel-based intensity standardization in the image processing, leading to slight discrepancies in 3D vertebral body geometry. *Fourth*, neither the skull nor the vertebral bodies were separated into cancellous and cortical bones. Future works may make the model more biofidelic by modeling cortical and cancellous bones separately. *Fifth*, CSF was modeled as a fluid-like incompressible soft material instead of as pure fluid. Future studies may continue to explore modeling CSF as a pure fluid and CSF-skull and CSF-brain interfaces as fluid–structure interactions. *Sixth*, we used four experimental studies to validate our model’s biomechanical responses. Nonetheless, it should be emphasized that other experimental studies are available, particularly based on sensor-based brain strain measurement (Hardy et al. 2001; Trosseille et al. 1992). *Seventh*, the model’s scalp thickness was thicker, and the CSF volume was larger, as we did not separate the intracranial blood vessels, and the meninges of CSF. As the mechanical response of the brain depends on both scalp thickness and CSF volume, some discrepancies between the model response and experimental data (Alshareef’s study) can be attributed to the differences in experimental and modeled CSF and scalp size. *Eighth*, we did not perform a formal mesh convergence analysis due to computational demands. A thorough mesh convergence analysis to determine optimal mesh sizes, especially for the finer brain hexahedral meshes, by simulating various injury or impact scenarios, warrants a separate study. *Ninth*, Neck ligaments were modeled using linear spring elements, which may limit the model’s ability to capture the nonlinear strain behavior of the ligaments. Although the neck structure with the current ligament definition has shown results that closely match the experimental results, future refinement of the model may include nonlinear and viscoelastic ligament models to enhance the utility of the neck model. *Tenth*, while our current validation focused on overall neck kinematics (C0–C7), we acknowledge the importance of assessing individual intervertebral motions—especially for the C1-C2 region, which plays a key role in head rotation. The C1-C2 joint in our model was constructed with appropriate articulation and soft tissues (ligaments and muscles) to allow realistic 3D motion. Future studies may compare the intervertebral motion patterns and biomechanical behavior of the model with those reported in in-silico cervical spinal models (Dong et al. 2024a, 2024b; Liu et al. 2024) and the experimental stereo-radiographic-based studies (Anderst et al. 2015; Chowdhury et al. 2017) to further evaluate the model's fidelity at specific cervical levels. *Eleventh,* while we evaluated brain displacement and strain at 24 receiver locations in the Alshareef’s study, the exact positions of these receivers were approximated based on the reported coordinates. Additionally, as our model’s brain shape was not exactly identical to the cadaver used by Alshareef's study, the location of some receivers was not perfectly matched. Despite these limitations, our developed head-neck model successfully reproduced the experimental results and provided valuable insights into the mechanical responses of the brain, both with and without the presence of neck structures.

### Conclusion

In this study, we present a subject-specific, MRI-derived head-neck FE model that includes detailed anatomical structures, such as scalp, skull, dura and pia mater, CSF, white and gray matter, cervical vertebrae (C1–C7), intervertebral disks, ligaments, and muscles. This unified approach allows us to investigate both brain intracranial responses and cervical spine mechanics within a single framework for studying TBI mechanisms under various accident and head injury scenarios that are commonly seen in occupational and military settings. Especially, the model can be used to evaluate the material and mechanical characterizations of helmets under various ballistic and non-ballistic mechanical impacts, head and neck injury risks in whiplash injuries that are commonly observed in car crashes, occupational falls, and mechanical load assessments for both neck and head implants. While the model shows potential for these applications, we have discussed areas for further refinements in the limitations section in order to enhance its utility in both research and clinical contexts.

## Supplementary Information

Below is the link to the electronic supplementary material.

Supplementary file1 (DOCX 103 KB)

## Data Availability

No datasets were generated or analysed during the current study.
