# Supplementary material for: An MRI-derived head-neck finite element model
Source: Biomech Model Mechanobiol. 2025 Oct 3;24(6):2195–215. doi: 10.1007/s10237-025-02013-x (PMC12618318; doi:10.1007/s10237-025-02013-x)
Supplement: Supplementary file 1 — Supplementary file1 (DOCX 103 KB) [file 10237_2025_2013_MOESM1_ESM.docx]

# Supplementary Materials

This supplementary document provides additional anatomical data, model comparisons, and figures referenced in the main manuscript.

## Table S1. Cervical muscles included in the model

List of cervical muscles with their classification (flexor/extensor) and anatomical origin and insertion points (based on Gray’s Anatomy (Standring 2021)).

| **Muscle Name** | **Flexor/Extensor** | **Origin** | **Insertion** |
| --- | --- | --- | --- |
| Obliquus capitis superior | Extensor | Transverse process of atlas (C1) | Occipital bone between superior and inferior nuchal lines |
| Superior longus colli | Flexor | Anterior tubercles of transverse processes of C3–C5 | Anterior tubercle of atlas (C1) |
| Rectus capitis major | Extensor | Spinous process of axis (C2) | Inferior nuchal line of occipital bone |
| Rectus capitis minor | Extensor | Posterior tubercle of atlas (C1) | Medial part of inferior nuchal line |
| Longus capitis | Flexor | Anterior tubercles of C3–C6 transverse processes | Basilar part of occipital bone |
| Rectus capitis anterior | Flexor | Lateral mass and root of transverse process of C1 | Basilar part of occipital bone |
| Rectus capitis lateralis | Flexor | Transverse process of atlas (C1) | Jugular process of occipital bone |
| Anterior scalene | Flexor | Anterior tubercles of C3–C6 transverse processes | Scalene tubercle of 1st rib |
| Middle scalene | Flexor | Posterior tubercles of C2–C7 transverse processes | Superior surface of 1st rib |
| Posterior scalene | Flexor | Posterior tubercles of C4–C6 transverse processes | External surface of 2nd rib |
| Sternocleidomastoid | Flexor | Manubrium (sternal head), medial clavicle (clavicular head) | Mastoid process and superior nuchal line |
| Longissimus capitis | Extensor | Transverse processes of C4–T4 | Mastoid process of temporal bone |
| Longissimus cervicis | Extensor | Transverse processes of T1–T5 | Posterior tubercles of C2–C6 transverse processes |
| Multifidus cervicis | Extensor | Articular processes of C4–C7 | Spinous processes 1–3 levels above origin |
| Semispinalis capitis | Extensor | Transverse processes of T1–T6 and C4–C7 articular processes | Occipital bone between superior and inferior nuchal lines |
| Semispinalis cervicis | Extensor | Transverse processes of T1–T6 | Spinous processes of C2–C5 |
| Splenius capitis | Extensor | Nuchal ligament, spinous processes of C7–T3 | Mastoid process and superior nuchal line |
| Splenius cervicis | Extensor | Spinous processes of T3–T6 | Transverse processes of C1–C3 |
| Levator scapulae | Extensor | Posterior tubercles of C1–C4 transverse processes | Superior angle and medial border of scapula |
| Obliquus capitis inferior | Extensor | Spinous process of axis (C2) | Transverse process of atlas (C1) |
| Trapezius | Extensor | External occipital protuberance, nuchal ligament, C7 spinous process | Lateral third of clavicle and acromion |

## Table S2. Neck ligaments included in the model

List of cervical ligaments with their anatomical attachments and primary functions (based on Gray’s Anatomy (Standring 2021)).

| **Ligament Name** | **Attachments** | **Function** |
| --- | --- | --- |
| Anterior longitudinal ligament | Anterior surfaces of vertebral bodies from occiput to sacrum | Limits hyperextension, supports intervertebral discs anteriorly |
| Posterior longitudinal ligament | Posterior surfaces of vertebral bodies inside vertebral canal | Limits hyperflexion, prevents posterior disc herniation |
| Ligamentum flavum | Between adjacent laminae from C1–C2 to L5–S1 | Limits abrupt flexion, assists spinal extension |
| Capsular ligament | Encapsulates facet joints between articular processes | Stabilizes and limits motion of facet joints |
| Interspinous ligaments | Connects adjacent spinous processes | Resists separation of spinous processes during flexion |
| Tectorial membrane | From body of C2 and posterior dens to basilar occiput | Stabilizes craniovertebral junction, limits flexion |
| Anterior atlanto-occipital ligament | From anterior arch of atlas to basilar occiput | Reinforces anterior atlanto-occipital joint, limits extension |
| Posterior atlanto-occipital ligament | From posterior arch of atlas to posterior foramen magnum | Limits flexion, protects vertebral artery and spinal cord |
| Anterior atlanto-axial ligament | Between anterior arch of atlas and body of axis | Reinforces atlanto-axial joint, limits extension |
| Posterior atlanto-axial ligament | Between posterior arch of atlas and lamina of axis | Limits flexion, protects spinal cord |
| Apical ligament | From apex of dens to anterior margin of foramen magnum | Minor stabilizer of dens to skull |
| Alar ligaments | From sides of dens to medial occipital condyles | Limit axial rotation and lateral flexion |
| Transverse ligament | Between medial tubercles of atlas lateral masses | Secures dens against anterior arch of atlas |
| Cruciate ligament of atlas | Transverse ligament with vertical superior and inferior bands | Stabilizes atlanto-axial joint and dens |

## Table S3. Comparison Between Our Model and Published Models

Comparison of key features, computational metrics, and anatomical details between the present model and other published head or head-neck finite element models.

| **Feature** | **Current Model** | **GHBMC (M50)** | **THUMS (AM50 V4-V7)** | **WHIM (V1.5/V2.0)** |
| --- | --- | --- | --- | --- |
| **MRI Derived (Head Geo)** | Full (Subject MRI) | Partial (MRI, CT) (Gayzik et al. 2011) | Partial (Primarily CT, some MRI) (Liang et al. 2025) | Full  (Zhao and Ji 2020) |
| **MRI Derived (Neck Geo)** | Full (Subject MRI) | Partial (MRI, CT) (Gayzik et al. 2011) | Partial (CT for bones, MRI for some muscle geo in ActiveTHUMS) (Iwamoto and Nakahira 2014) | Absent (Zhao and Ji 2020) |
| **Brain Element Count (Approx.)** | ~600k | ~164k-211k (Lyu et al. 2022) | ~50k (Iwamoto et al. 2007) | ~203k (V1.5/V2.0) (Zhao and Ji 2020) |
| **Avg. Brain Element Size (mm)** | ~1.3 | ~2 (Li et al. 2021) | ~3-7 (Shigeta et al. 2009) | ~1.8 (V1.5/V2.0) (Zhao and Ji 2020) |
| **Brain Element Type** | Hexahedral | Hexahedral (Lyu et al. 2022) | Hexahedral (Iwamoto and Nakahira 2014) | Hexahedral (C3D8R) (Zhao and Ji 2020) |
| **Neck Muscles** | Active (Hill-type) | Active (3D passive + 1D active) (Gayzik et al. 2011) | Active (Hill-type; 1D or 1D+Solid) (Chawla et al. 2005) | Absent (Zhao and Ji 2020) |
| **Key Validation Basis** | Kinematics (NBDL), ICP (Nahum), Brain Motion (Alshareef), Cervical Strain (Ito) | PMHS Kinematics, ICP, Strain, Sled tests (Decker et al. 2017; Gayzik et al. 2011; Lyu et al. 2022) | PMHS (Components, Full Body, Sled etc) | PMHS (Strain, Pressure, Displacement), *In vivo* motion (Zhao and Ji 2020) |
| **Typical Sim. Time Example** | 12h/128core (200 ms) | 3.5h/36core (Head/Neck); 8.4min/ms (M50-O) (Decker et al. 2017) | 16h/4CPU (200ms full body) (Iwamoto et al. 2015) | 30min/15CPU+GPU (100ms impact) (Zhao and Ji 2020) |

Continued Table S3

| **Feature** | **ADAPT** | **SUFEHM** | **SIMon (Next-Gen)** | **Imperial College Model** |
| --- | --- | --- | --- | --- |
| **MRI Derived (Head Geo)** | Full (ICBM152 MRI Atlas) (Li et al. 2021) | Partial (Digitized Skull, MRI/DTI for brain) (Deck and Willinger 2008) | CT  (Bandak et al. 2001) | Full (Subject MRI) (Ghajari et al. 2017) |
| **MRI Derived (Neck Geo)** | Generally Absent (Li et al. 2021) | Generally Absent/ATD/THUMS (Deck and Willinger 2008; MAYER et al.) | Absent (Bandak et al. 2001) | Absent (Ghajari et al. 2017) |
| **Brain Element Count (Approx.)** | Up to 4.4M (entire head) (Li et al. 2021) | 5,320 (brain) (Kang et al. 1997) | Part of 40.7k solids (Takhounts et al. 2008) | ~1M (head, mostly brain) (Ghajari et al. 2017) |
| **Avg. Brain Element Size (mm)** | 0.5-2.5 (Li et al. 2021) | 1.14-7.73 (Kang et al. 1997) | Not Specified (Takhounts et al. 2008) | ~1.5 (Ghajari et al. 2017) |
| **Brain Element Type** | Hexahedral (Li et al. 2021) | Hexahedral (Deck and Willinger 2008) | Hexahedral (Takhounts et al. 2008) | Hexahedral (Ghajari et al. 2017) |
| **Neck Muscles** | Absent (Li et al. 2021) | Passive (ATD/THUMS) (Deck and Willinger 2008) | Absent (Takhounts et al. 2008) | Absent (Ghajari et al. 2017) |
| **Key Validation Basis** | PMHS (Relative Motion, Strain, ICP) (Li et al. 2021) | PMHS (ICP, Brain Motion), Real-World Cases, Skull Fracture (Deck and Willinger 2008) | Motion, Pressure (Takhounts et al. 2008) | PMHS (Brain Displacement) (Ghajari et al. 2017) |
| **Typical Sim. Time Example** | 22h/256CPU (100ms impact) (Li et al. 2021) | Not Specified | 10h (150ms) (Takhounts et al. 2008) | 4h/32CPU  (50ms impact) (Duckworth et al. 2022) |

## Figure S1. Peak axial strain in neck muscles

Bar plot shows representative peak axial strain values in neck muscles from NBDL simulated scenario.


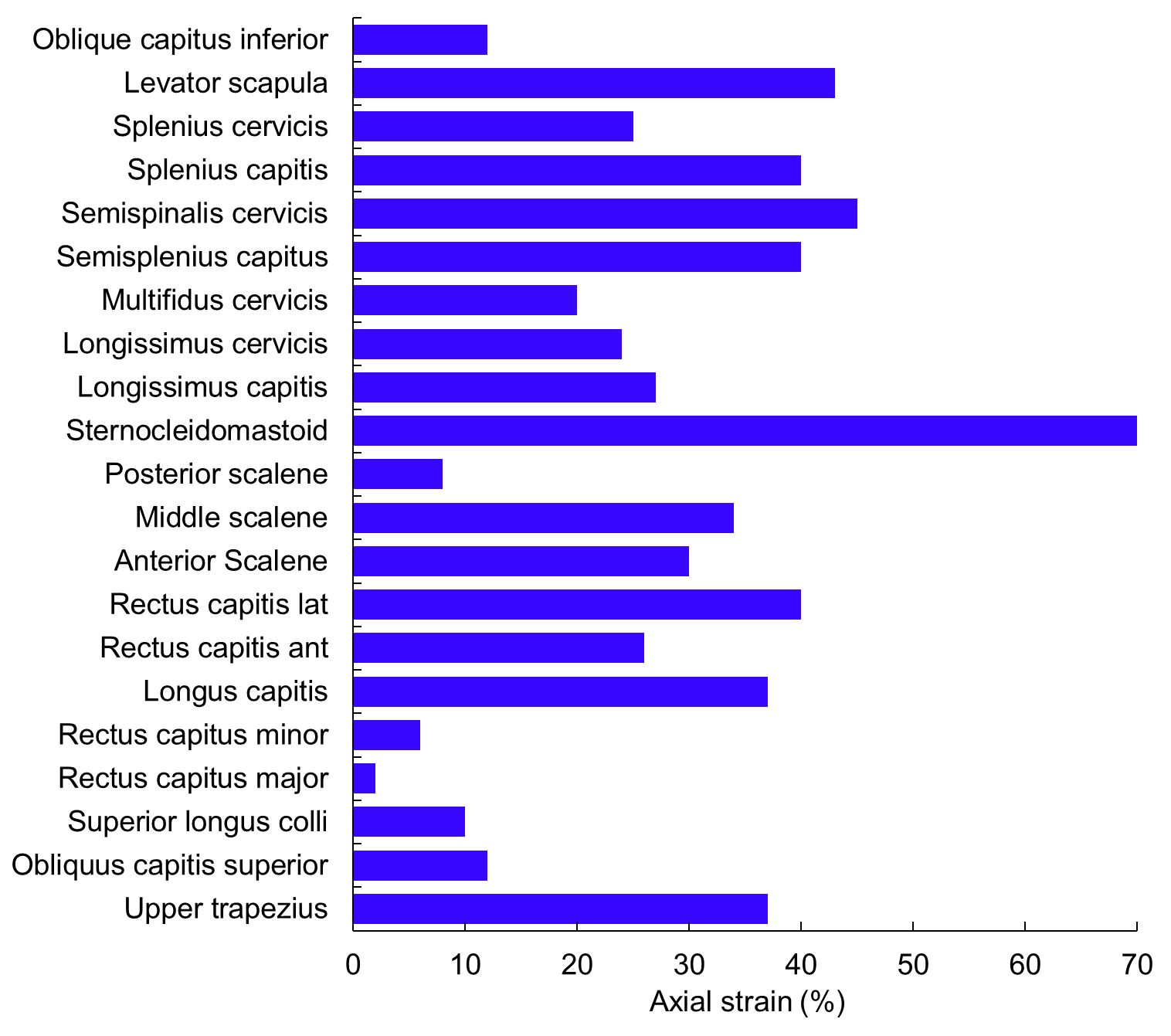


**References**

Bandak F, Zhang A, Tannous R, DiMasi F, Masiello P, Eppinger R (2001) Simon: a simulated injury monitor; application to head injury assessment. SAE Technical Paper,

Chawla A, Mukherjee S, Mohan D, Jain S Validation of the cervical spine model in THUMS. In: Proceedings of the 19th International Technical Conference on the Enhanced Safety of Vehicles Washington DC USA, 2005. Citeseer,

Deck C, Willinger R (2008) Improved head injury criteria based on head FE model International Journal of Crashworthiness 13:667-678

Decker W, Koya B, Davis ML, Gayzik FS (2017) Modular use of human body models of varying levels of complexity: validation of head kinematics Traffic injury prevention 18:S155-S160

Duckworth H, Azor A, Wischmann N, Zimmerman KA, Tanini I, Sharp DJ, Ghajari M (2022) A finite element model of cerebral vascular injury for predicting microbleeds location Frontiers in bioengineering and biotechnology 10:860112

Gayzik FS, Moreno DP, Vavalle NA, Rhyne AC, Stitzel JD Development of the global human body models consortium mid-sized male full body model. In: International workshop on human subjects for biomechanical research, 2011. National Highway Traffic Safety Administration Washington, DC, USA,

Ghajari M, Hellyer PJ, Sharp DJ (2017) Computational modelling of traumatic brain injury predicts the location of chronic traumatic encephalopathy pathology Brain 140:333-343

Iwamoto M, Nakahira Y A preliminary study to investigate muscular effects for pedestrian kinematics and injuries using active THUMS. In: Proceedings of the IRCOBI conference, IRC-14–53, Berlin, Germany, 2014. pp 444-460

Iwamoto M, Nakahira Y, Kimpara H (2015) Development and validation of the total human model for safety (THUMS) toward further understanding of occupant injury mechanisms in precrash and during crash Traffic injury prevention 16:S36-S48

Iwamoto M, Nakahira Y, Tamura A, Kimpara H, Watanabe I, Miki K Development of advanced human models in THUMS. In: Proc. 6th European LS-DYNA Users' Conference, 2007. pp 47-56

Kang H-S, Willinger R, Diaw BM, Chinn B (1997) Validation of a 3D anatomic human head model and replication of head impact in motorcycle accident by finite element modeling SAE transactions:3849-3858

Li X, Zhou Z, Kleiven S (2021) An anatomically detailed and personalizable head injury model: Significance of brain and white matter tract morphological variability on strain Biomechanics and modeling in mechanobiology 20:403-431

Liang Z, Wu K, Tian T, Mo F (2025) Human head–neck model and its application thresholds: a narrative review International Journal of Surgery 111:1042-1070

Lyu D, Zhou R, Lin C-h, Prasad P, Zhang L (2022) Development and validation of a new anisotropic visco-hyperelastic human head finite element model capable of predicting multiple brain injuries Frontiers in Bioengineering and Biotechnology 10:831595

MAYER C, DECK C, LUCE H, DE GUESELLE P, WILLINGER R COUPLING OF STRASBOURG UNIVERSITY HEAD MODEL TO THUMS HUMAN BODY FE MODEL: VALIDATION AND APPLICATION TO AUTOMOTIVE SAFETY IPEK H

Shigeta K, Kitagawa Y, Yasuki T (2009) Development of next generation human FE model capable of organ injury prediction Proceedings of the 21st annual enhanced safety of vehicles:15-18

Takhounts EG et al. (2008) Investigation of traumatic brain injuries using the next generation of simulated injury monitor (SIMon) finite element head model. SAE Technical Paper,

Zhao W, Ji S (2020) Displacement-and strain-based discrimination of head injury models across a wide range of blunt conditions Annals of biomedical engineering 48:1661-1677
